# Supplementary material for: Folding pathway of a discontinuous two-domain protein
Source: Nat Commun. 2024 Jan 23;15:690. doi: 10.1038/s41467-024-44901-3 (PMC10805907; doi:10.1038/s41467-024-44901-3)

Supplementary Materials for

Folding pathway of a discontinuous two-domain protein

Authors

Ganesh Agam[1,†], Anders Barth[1,‡] and Don C. Lamb[1]*

Affiliations

[1]Department of Chemistry, Center for NanoScience, Nanosystems Initiative Munich (NIM), and Center for Integrated Protein Science Munich (CiPSM), Ludwig-Maximilians University Munich, Munich, Germany.

[†]Current address: MRC Laboratory of Molecular Biology, Francis Crick Avenue, Cambridge Biomedical Campus, Cambridge, CB2 0QH, UK

[‡]Current address: Department of Bionanoscience, Kavli Institute of Nanoscience Delft, Delft University of Technology, 2629HZ, Delft, The Netherlands.

* Corresponding author: d.lamb@lmu.de

This PDF file contains:

Supplementary Notes 1-7
Figs. S1 to S18
Tables S1 to S9
Source Data files: Unprocessed SDS gel images

TABLE OF CONTENTS

SUPPLEMENTARY NOTES 4

SUPPLEMENTARY NOTE 1. CHARACTERIZATION OF A PREVIOUSLY KNOWN HYSTERESIS IN THE FOLDING OF DM-MBP...................... 4

SUPPLEMENTARY NOTE 2. THE INTERMEDIATE POPULATION IS ALSO PRESENT IN THE CTD AND N-C INTERFACE 5

SUPPLEMENTARY NOTE 3. KINETIC ANALYSIS OF REFOLDING FOR THE N-C INTERFACE 5

SUPPLEMENTARY NOTE 4. FILTERED FCS ANALYSIS OF THE NTD, CTD AND N-C INTERFACE 6

SUPPLEMENTARY NOTE 5. THE DYNAMIC PHOTON DISTRIBUTION ANALYSIS OF THE NTD, CTD AND THE N-C INTERFACE 7

SUPPLEMENTARY NOTE 6. CHARACTERIZATION OF THE 3C FRET CONSTRUCT... 7

SUPPLEMENTARY NOTE 7. MOLECULAR DYNAMIC SIMULATIONS OF MBP AND DM-MBP UNFOLDING.. 8

SUPPLEMENTARY FIGURES... 10

FIG. S1. UNFOLDING AND REFOLDING OF MBP 10

FIG. S2. DATA ANALYSIS AVAILABLE FROM MFD-PIE MEASUREMENTS USED FOR ALL 2C SMFRET EXPERIMENTS. 11

FIG. S3. 2C UNFOLDING/REFOLDING SMFRET MEASUREMENTS OF THE NTD, CTD AND THE N-C INTERFACE.............................. 12

FIG. S4. QUENCHING OF ALEXA647 OBSERVED DURING THE MBP REFOLDING STUDIES.. 14

FIG. S5. EQUILIBRIUM UNFOLDING-REFOLDING CURVES OF WT-MBP 15

FIG. S6. ANALYSIS OF THE DONOR FLUORESCENCE LIFETIME FOR DOUBLE-LABELED MOLECULES OF DM-MBP FROM 2C FRET MEASUREMENTS ON THE NTD, CTD AND THE N-C INTERFACE.. 17

FIG. S7. CONSERVATION OF CONFORMATIONAL DYNAMICS USING A DIFFERENT ACCEPTOR IN DM-MBP REFOLDING MEASUREMENTS. 18

FIG. S8: FOLLOWING THE REFOLDING OF DM-MBP IN 0.3 M GUHCL OVER 3 DAYS 19

FIG. S9. ARRHENIUS PLOT FOR THE REFOLDING RATES OF THE NTD CONSTRUCT AT DIFFERENT TEMPERATURES MEASURED BY SMFRET. 20

FIG. S10. CONFORMATIONAL DYNAMICS QUANTIFIED USING THE DYNAMIC PHOTON DISTRIBUTION ANALYSIS 22

FIG. S11. EXEMPLARY GLOBAL FILTERED FCS ANALYSIS FOR THE NTD REFOLDING IN 0.3 M GUHCL. 23

FIG. S12. THE P298C POSITION IS INACCESSIBLE FOR CYSTEINE-MALEIMIDE LABELING WHEN MALTOSE IS BOUND TO DM-MBP...... 24

FIG. S13. 3C SMFRET ANALYSIS OF DM-MBP USING MFD-PIE 26

FIG. S14 FUNCTIONAL ASSAY OF REFOLDED DM-MBP LABELED WITH ATTO488, ATTO565, AND ALEXA647 27

FIG. S15. 2C SMFRET CONTROL MEASUREMENTS FOR THE 3C SMFRET MEASUREMENTS 30

FIG. S16. CORRELATIONS FOUND BY 3C FRET IN FOLDED AND AN INTERMEDIATE POPULATION DURING THE FOLDING OF DM-MBP 31

FIG. S17. REPEATS OF MD SIMULATIONS PERFORMED ON WT-MBP AND DM-MBP FOR TEMPERATURE INDUCED UNFOLDING...... 32

FIG. S18. CHARACTERIZATION OF ASSISTED REFOLDING OF DM-MBP BY THE GROEL/ES CHAPERONINS. 34

SUPPLEMENTARY TABLES **35**

TABLE S1. THE REFOLDING RATES AND HALF-LIFE ($T_{1/2}$) OF REFOLDING FOR THE VARIOUS MBP MUTANTS. 35

TABLE S2. FÖRSTER DISTANCE AND CORRECTION FACTORS USED FOR THE VARIOUS COMBINATIONS OF DYE-PAIRS INVESTIGATED IN THIS STUDY. 36

TABLE S3. COMPARISON OF DISTANCES CALCULATED FROM THE ACCESSIBLE VOLUME (AV) AND 2C FRET EXPERIMENTS PERFORMED FOR ALL THE THREE DOUBLE-CYSTEINE DOMAIN MUTANTS OF DM-MBP LABELED WITH THE ATTO532-ALEXA647 DYE-PAIR. 37

TABLE S4. RESULTS FROM THE DONOR LIFETIME ANALYSIS OF ALL THREE 2C CONSTRUCTS OF DM-MBP LABELED WITH ATTO532-ALEXA647. 38

TABLE S5. RESULTS FROM THE FILTERED FCS ANALYSIS FOR ALL THE THREE 2C CONSTRUCTS OF DM-MBP LABELED WITH ATTO532-ALEXA647. 40

TABLE S6. RESULTS FROM THE DYNAMIC PDA FOR ALL THE THREE 2C DM-MBP CONSTRUCTS LABELED WITH ATTO532-ALEXA647.42

TABLE S7. FLUORESCENCE LIFETIME ANALYSIS OF ALEXA647 FOR TWO-COLOR DM-MBP CONSTRUCTS. 43

TABLE S8. FLUORESCENCE LIFETIMES OF ALEXA647 FOR TRIPLE-LABELED MOLECULES OF DM-MBP (52PRK-175C-298C). 43

TABLE S9. STEADY-STATE AND TIME-RESOLVED ANISOTROPIES OF DM-MBP CONSTRUCTS OF NTD, CTD AND N-C INTERFACE MEASURED WITH ATTO532 AND ALEXA647. 45

SOURCE DATA FILES: UNPROCESSED SDS GEL IMAGES (FIGURE S12) **46**

Supplementary Notes

Supplementary Note 1. Characterization of a previously known hysteresis in the folding of DM-MBP

We characterized the denaturation/refolding of DM-MBP using intrinsic tryptophan fluorescence taking advantage of the eight tryptophan residues present in DM-MBP (**Fig. 1a**) (PDB ID:1OMP) (*26*). Burial of solvent exposed hydrophobic tryptophan residues inside the protein core upon folding/refolding results in an increase in the tryptophan fluorescence. We also compared the tryptophan fluorescence of denaturation/refolding of WT-MBP. When MBP is completely unfolded in 3 M guanidine-hydrochloride (GuHCl), the tryptophan fluorescence is reduced by ~2.5-fold as compared to the native protein. We first performed equilibrium measurements of unfolding and refolding by titrating with different GuHCl concentrations. The MBP concentration for these experiments was kept at 40 nM (**Fig. S1a**). In the case of unfolding, when native MBP was denatured with increasing concentrations of denaturant, both WT and DM-MBP starts to unfold above 0.6 M GuHCl. Complete unfolding was observed above 1 M GuHCl. Strikingly, in contrast to WT, which follows a similar trend during refolding, DM-MBP only refolds from the denatured state at concentrations much less than 0.6 M GuHCl. The hysteresis in formation of the native state with respect to unfolding has been attributed to kinetic traps present in the folding landscape of the protein (*76*). The same observation was made by Chakraborty et al. 2010 (*25*), who hence proposed a kinetically trapped state in DM-MBP. Notably, DM-MBP completely refolds to its native state below a final concentration of 0.1 M GuHCl in refolding buffer (**Fig. S1a**). Next, we measured the kinetics of refolding. Denatured WT and DM-MBP in 3 M GuHCl was allowed to refold after a 75-fold dilution to a final concentration of 40 nM MBP in 40 mM GuHCl. The refolding half-time ($t_{1/2}$) for WT and DM-MBP was found to be 0.29±0.07 and 24.7±2.7 min with a refolding rate of 2.4±0.5 and 0.028±0.003 min^{-1} respectively (**Fig. S1b**, **Table S1**).

The delayed refolding, which gives rise to the hysteresis effect, might be caused by inter-molecular interactions (i.e. aggregation) of the unfolded peptide chain due to the high degree of exposed hydrophobic residues. To exclude this possibility, we used 40 nM protein concentration as we did for the tryptophan fluorescence assays. To verify that there is no aggregation at these concentrations, we performed fluorescence cross-correlation spectroscopy (FCCS) experiments. FCCS, in combination with pulsed interleaved excitation (PIE) is highly sensitive to the presence of any interaction between molecules labeled with different fluorophores (*61*), giving rise to a cross-correlation signal. As a positive control, we measured double-stranded DNA labeled with

both Atto532 and Atto647N, which gives rise to a significant cross-correlation signal (red curve in **Fig. S1c**). A single-cysteine mutant of DM-MBP (A52C) labeled with either Atto532 or Alexa647 (500 - 1000 nM) were unfolded in 3 M GuHCl for 30 min at 50°C, mixed in equal amounts and diluted to allow refolding in 0.1 M GuHCl at a final protein concentration of 40 nM. No cross-correlation amplitude was detected indicating that no aggregation occurred during refolding over the course of 60 minutes (green, magenta and yellow curves in **Fig. S1c**). As a negative control, a mixture of freely-diffusing dyes was also measured, which shows no cross-correlation signal (blue curve in **Fig. S1c**). The observed hysteresis effect and refolding rate of DM-MBP are in good agreement with previous reports (*24*, *25*)

Supplementary Note 2. The intermediate population is also present in the CTD and N-C interface

Having characterized the intermediate population in the NTD, we investigated whether refolding of the CTD and formation of the N-C interface also exhibit the same hysteresis. To monitor the CTD conformation, we made use of the previously characterized double-cysteine mutant of DM-MBP (175C-298C) (**Fig. 1a**, **Fig. S3a**) (*24*). Equilibrium unfolding and refolding curves were recorded using 2C smFRET measurements on the CTD labeled with the same dye-pair as for the NTD (Atto532 and Alexa647) (**Fig S3d**). The native and completely unfolded states of the CTD have similar donor-acceptor separations as for the native and completely unfolded state of NTD, respectively (**Table S3**). Waterfall plots of the FRET efficiency versus the titrated GuHCl concentration for refolding and unfolding measurements show a similar trend as for the NTD and refolding has a similar intermediate population with a FRET efficiency of 0.6 (**Fig. S3d**, **Fig. 2b**). Kinetic experiments on CTD refolding yielded a $t_{1/2}$ of 19±8 min (**Fig. S3e**). A similar observation was made for equilibrium and kinetic measurements on the N-C interface (52C-175C) (**Fig. S3f-g**). For the N-C interface, the intermediate population is more compact with a FRET efficiency of 0.85 (**Fig. 2b**). The structure of the N-C interface is well defined in the native state, having a small width of 2.8 Å for an inter-dye distance of 44.8 Å as determined by PDA. The AV simulations predict an inter-dye separation of 40.0 Å (**Table S3**, **Fig. S3a**). The unfolded state has a distance of 74.9 Å, comparable to the NTD and CTD unfolded states (**Table S3**). The kinetics of refolding are similar to that of the other mutants and to the unlabeled protein, which implies the functionality of the both the labeled CTD and N-C interface constructs (**Fig. S3e,g**).

Supplementary Note 3. Kinetic analysis of refolding for the N-C interface

Due to the similar FRET efficiencies of the folded and the kinetically trapped state in the N-C interface construct, a kinetic analysis based on the FRET efficiency histograms alone was not possible. Hence, we made use of the observation that the fluorescence lifetime of Alexa647 is different in the unfolded and the refolded protein. This phenomenon was also observed in CTD construct where it is more visible than for the N-C interface (**Fig. S4a**). Due to steric restriction, the cis-trans isomerization of Alexa647 is hindered in the unfolded state, a phenomenon that is typical for cyanine-based dyes. Hence, the dye exhibits a higher lifetime of ~1.7 ns for the unfolded state, but has a lifetime of ~1.2 ns in the refolded protein, close to that of free dye in water (**Fig. S4a-b** and **Table S7**). Despite the different acceptor lifetimes, the measured anisotropy for Alexa647 remained below ~0.25 for all constructs (**Table S9**). To ensure that the observed intermediate population was not an artifact arising from the acceptor, we measured refolding with a different acceptor, Atto647N where the same dynamic intermediate population was observed (**Fig. S4c**, **Fig. S7**). Appropriate corrections were made to account for the differences in the acceptor quantum yield for the different populations for all constructs labeled with Alexa647 (**Fig. S4d** and **Table S2**).

Supplementary Note 4. Filtered FCS analysis of the NTD, CTD and N-C interface

A burstwise filtered FCS analysis correlates the signal fluctuation in selected sub-populations based on their FRET efficiency, taking the advantage of filters available such as fluorescence lifetime, wavelength, and polarization. The analysis is sensitive to fluctuations in intensity down to microseconds. We analyzed the refolding measurements for all the three constructs to investigate the dynamic rates present in DM-MBP during the refolding reaction. The results are summarized in **Table S5**.

A global analysis of all the measurements from the same construct recovered two relaxation rates on the order of 4-10 μs and 180-350 μs. The fast relaxation time (4-10 μs) can be related to the fast fluctuations between the labeled positions in an unfolded polypeptide (i.e. the peptide reconfiguration time). In light of previous findings on short polypeptides with relaxation times on the order of 50-200 ns (*34*), this highlights the complexity in the dynamics of an unfolded state of a large polypeptide chain. The dynamics are mainly governed by the rugged free energy surface but a full understanding of the dynamics will require further investigations. The second relaxation time (180-350 μs) is the same as that determined using dynamic PDA and can be related to large conformational fluctuations of the polypeptide as it undergoes refolding.

Supplementary Note 5. The Dynamic Photon Distribution Analysis of the NTD, CTD and the N-C interface

The dynamic PDA analysis routine quantifies the rates at which subpopulations interconvert during the burst duration of a few milliseconds. The distance distributions are modeled based on the raw photon counts to quantify the broadening of the smFRET histograms beyond that of shot-noise. The robustness of the fit is increased by generating FRET efficiency histograms (or proximity ratio histograms) of different time resolutions between ca 0.2 and 1.5 milliseconds and globally fitting the histograms. The distances, widths and kinetic rates can be extracted from the analysis to determine the microscopic rates between the states. The FRET efficiency of different states was defined using the donor lifetimes **(Table S4).**

Table S6 summarizes the microscopic rates for conformational fluctuations between the collapsed compact (C) and unfolded state (U). For all three constructs, the apparent unfolding rate ($k_{C\rightarrow U}$) increases with increasing GuHCl concentration **(Fig. 3B, Fig. S10, Table S6).** This is consistent with a loss of the natively stabilized contacts by the increasing amount of GuHCl in solution. In contrast, there are striking differences in the apparent folding rates ($k_{U\rightarrow C}$) of the three constructs. For the CTD and N-C interface, the transition rates remained unchanged between 2-3 ms^{-1} for the CTD and between 3.3 and 3.9 ms^{-1} for the N-C interface throughout all the refolding curves with the exception of refolding of the N-C interface in 0.1 M GuHCl. As GuHCl destabilizes native contacts, the refolding rates are expected to be independent of GuHCl concentration. The reduction in the refolding kinetics of the N-C interface at 0.1 GuHCl is most likely due to higher friction of the polypeptide chain in both the nearly folded and unfolded conformations. For the NTD, a more complicated pattern is observed. The apparent unfolding rates ($k_{C\rightarrow U}$) are between 2-3 ms^{-1} for lower GuHCl concentrations and then slow down to 0.6 - 0.9 ms^{-1} at higher GuHCl concentrations. One possible explanation is that the hydrophobic core in the NTD defined by the residues 8-21, 22-24 and 45-63 is needed to stabilize the folded state (*22*). Disruption of the hydrophobic core by GuHCl also decreases the refolding transition monitored with the NTD FRET construct. The similar relaxation times for the NTD and the N-C interface indicates cooperativity in the folding process.

Supplementary Note 6. Characterization of the 3C FRET construct

We measured the native, denatured and refolded state of 3C-labeled DM-MBP. Due to our specific labeling strategy, the FRET efficiency between the blue and green dyes (BG) reports on the NTD conformation, the FRET efficiency between the green and red dyes (GR) corresponds

to the CTD conformation and the FRET efficiency between the blue and red dyes (BR) monitors the N-C interface (**Fig. 4a**). The 3C smFRET measurement of triple-labeled DM-MBP in the native state showed a high FRET peak at ~0.9 for all the three FRET efficiencies. Refolded DM-MBP showed the same FRET efficiencies as for the native state for all three FRET pairs, confirming the correct refolding of the triple-labeled DM-MBP (**Fig. S15d-e**). When DM-MBP was denatured in 3 M GuHCl, the FRET efficiency BG and BR was found to be centered at a value of ~0, while the FRET efficiency GR was at ~0.35. To confirm the specific labeling of the dyes and to validate the results with the dyes used for the 3C FRET experiments, we additionally measured double-cysteine mutants probing NTD, CTD and N-C interface separately with 2C smFRET measurements using the respective dye-pairs (**Fig. S15**).

We performed the equilibrium unfolding and refolding measurements using the triple-labeled DM-MBP. The results are consistent with that of the 2C smFRET experiments (**Fig. 1b** and **Fig S3d,f**) showing a similar unfolding trend (**Fig. 4b**). Next, we probed the equilibrium refolding, obtaining a similar trend as compared to the 2C FRET experiments (**Fig. 4c**, **Fig. 1b** and **Fig 2a-b**). For the histogram of the FRET efficiency between the blue and green dye, an intermediate population with E_{BG} = 0.7 was evident at concentrations of 0.1-0.5 M GuHCl (**Fig. 4**), in agreement with the intermediate population seen for the NTD (**Fig. 1b**) and the 2C FRET controls using the blue-green dye pair (**Fig. S15d-e**, left panels). Similarly, an intermediate population was present for the CTD and N-C interface with E_{GR} ~0.8 and E_{BG}~0.7, respectively (**Fig. 4C**), which was confirmed by 2C FRET controls with the green-red and blue-red dye pairs, respectively (**Fig. S15d-e**, middle and right panels).

Supplementary Note 7. Molecular Dynamic simulations of MBP and DM-MBP unfolding

It is currently not possible to simulate the folding trajectory of a slowly folding protein starting from the denatured state on a realistic time scale. However, unfolding can be induced by increasing the temperature, giving insights into the folding pathways of proteins in a reverse manner (*77*, *78*). In the case of WT-MBP, which folds/refolds within a minute, it was shown that the NTD folds before the CTD (*23*). Here, for DM-MBP, we found the same folding order but delayed on the timescale of ~20-30 min (**Fig. S1b**) (*21*). To investigate the origin of these differences, we simulated the temperature-induced unfolding of WT-MBP and DM-MBP with all atom MD simulations starting from the native conformation (PDB: 1OMP). Simulations were carried out for 2 μs time at 450 K after equilibrating the protein for 2 μs at 400 K. Indeed, the unfolding trajectory for WT-MBP shows that the majority of the secondary structures in the CTD unfolds first while,

for the NTD, parts of the secondary structure are preserved until the end of the simulation (**Fig. S17a**). These regions include two alpha-helices (15-25 amino acid residues and 280-286 amino acid residues) and two-anti-parallel beta sheets (**Fig. S17c**). This suggests that, when WT-MBP folds, these two helices and two- beta sheets are the first structural elements in the NTD and the rest of the CTD folds later. This folding nucleus is disrupted by the double mutations introduced in the DM-MBP. We ran two repeats for WT-MBP for unfolding simulations directly at 450 K for 2 μs without the initial 2 μs equilibration run at 400 K (**Fig. S17a**), which showed the same trend of unfolding events for the NTD and CTD. These results agree with the folding order measured in previous studies on WT-MBP (*22*, *23*). For DM-MBP, one global unfolding event was observed, and no major secondary structure was preserved at the end of the simulation (**Fig. S17b**). Repeating the simulations run directly at 450 K also led to complete unfolding in a single step within 300 ns. This clearly suggests a decreased stability as the native contacts were lost more quickly in DM-MBP.

Supplementary Figures

Fig. S1

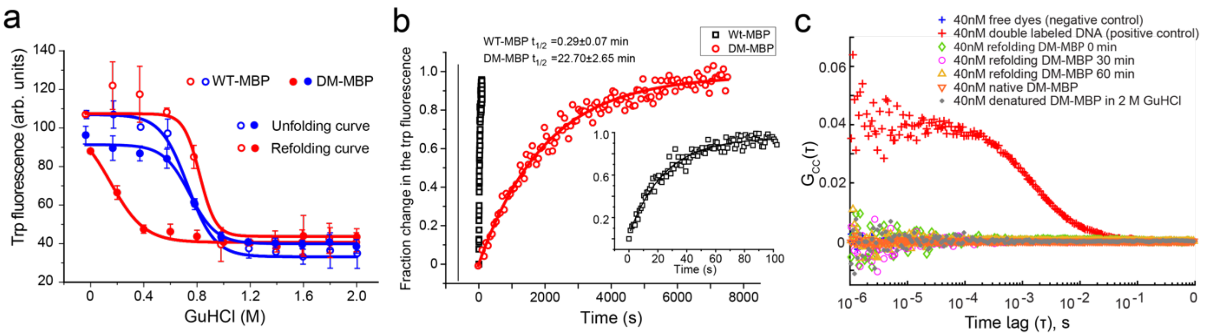

Fig. S1. Unfolding and refolding of MBP

(a) Equilibrium unfolding and refolding experiments at different GuHCl concentrations of WT-MBP (open circles) and DM-MBP (closed circles) measured using tryptophan fluorescence. To record the unfolding curve (blue), steady state tryptophan fluorescence of ~40 nM native MBP was measured after 20 hr in the respective GuHCl concentration at 22 °C. For the refolding curve (red), 2 μM of MBP was first denatured in 3 M GuHCl for 1 hr at 50°C, diluted 50-fold and incubated for 3-4 hrs at the indicated final GuHCl concentrations for before measuring the steady state tryptophan fluorescence. The given error is the standard deviation from at least three independent titrations. Each curve was fitted with a Boltzmann function (see **Supplementary Note 1**).

(b) The kinetics of WT-MBP (black squares) and DM-MBP (red open circles) refolding monitored by the increase in tryptophan fluorescence. The initial fluorescence at time t=0 was subtracted from the subsequent data points. 3 μM MBP was denatured in 3 M GuHCl for 1 hr at 50 °C before being diluted 75-fold in buffer A to start the refolding reaction (at t=0, the final concentrations were ~40 nM of MBP and 40 mM of GuHCl). Data were fitted using a single exponential function. The fit to the WT-MBP refolding kinetics is shown in the inset for the clarity. The presented data is from a single measurement representative of three independent measurements.

(c) FCCS measurements of a mixture of 20 nM Atto532 labeled DM-MBP (A52C) and 20 nM Alexa647 labeled DM-MBP undergoing refolding after 0 min, 30 min and 60 min (green, magenta and yellow curves, respectively). 500-1000 nM of DM-MBP (A52C) was first denatured in 3 M GuHCl for 1 hr at 50 °C. The sample was then diluted first serially in 3 M GuHCl before the final dilution to achieve the nanomolar concentrations of the labeled protein. Atto532 and Atto647N labeled DNA served as a positive control for a cross-correlation signal (red curve). Freely diffusing Atto532 and Atto655 dyes were measured as a negative control (blue curve). We also performed the FCCS analysis under native (orange curve) and denaturing (grey curve) conditions.

Fig. S2

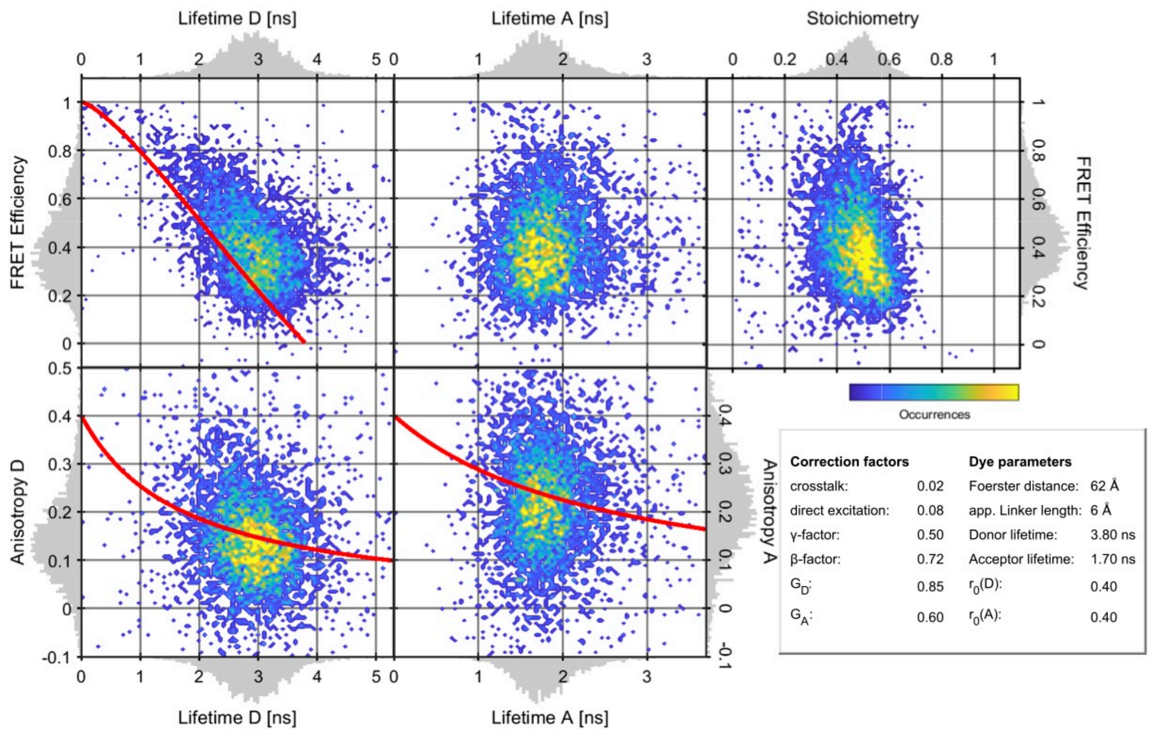

Fig. S2. Data analysis available from MFD-PIE measurements used for all 2C smFRET experiments.

A representative all-in-one plot for 2C smFRET measurements using multi-parameter fluorescence detection with pulsed interleaved excitation (MFD-PIE) of NTD refolding performed in 0.9 M GuHCl. Each burst represents a single molecule. The upper left panels show the relationship between FRET efficiency and burst-wise donor lifetime (Lifetime D in ns) and acceptor lifetime (Lifetime A in ns). The ideal relationship between the FRET efficiency and donor lifetime is highlighted by the red line for static samples. The upper right panel shows a stoichiometry (S) of ~0.5 (typical for double labeled molecules) and the FRET efficiency for the analyzed molecules. The lower panels depict the burst-wise anisotropy values for donor (D) and acceptor (A) fluorophores with their respective lifetimes. Red lines are fits to the Perrin equation. The applied correction factors are shown in the lower right panel and given in **Table S2**.

Fig. S3

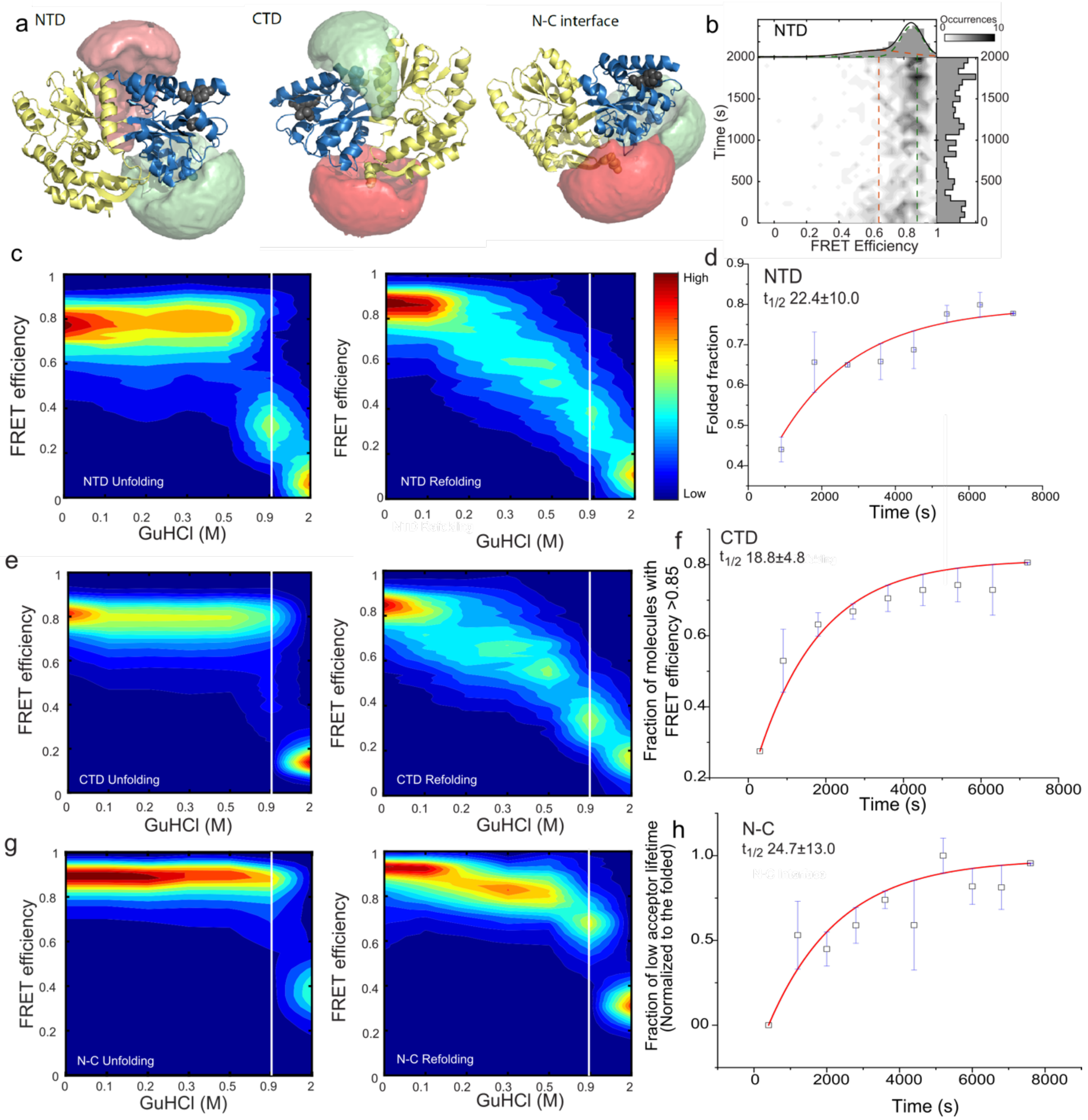

Fig. S3. 2C unfolding/refolding smFRET measurements of the NTD, CTD and the N-C interface

(a) Accessible volume calculations using the MBP structure (PDB ID:1OMP), and Atto532 and Alexa647 dyes. Calculations were performed with this one dye combination for the NTD, CTD and N-C interface constructs. Left panel: The NTD labeled at positions A52 (Atto532) and P298 (Alexa647), Middle panel: The CTD labeled at positions K175 (Alexa647) and P298 (Atto532),

Right panel: The N-C interface labeled at positions A52 (Atto532) and K175 (Alexa647) (**Table S3**).

(b) Kinetics of NTD refolding in 0.1 M GuHCl was analyzed for the initial 2000 s of a single-molecule burst analysis experiment.

(c-d) Equilibrium and kinetic analyses of NTD unfolding and refolding. **(c)** Waterfall plots of FRET efficiency versus denaturant. The sample was incubated for 2 hours before the start of the measurement to establish a pseudo equilibrium. (d) The refolding kinetics for the NTD in 0.1 M GuHCl was calculated by assaying the increase in the folded fraction (0.85 FRET efficiency) from panel B as a function of time. The red curve is a mono-exponential fit to the data (black). The half-life ($t_{1/2}$) for the refolding process is 22.4 ± 10.0 min (**Table S1**). The curve is the average of three independent experiments. Errors are the SD of the mean.

(e-f) Equilibrium and kinetic analyses of CTD unfolding and refolding. **(e)** Waterfall plots of FRET efficiency versus denaturant concentration. The sample was incubated for 2 hours before the start of the measurement to establish a pseudo equilibrium. **(f)** A kinetic analysis of the smFRET histograms for CTD refolding. The curve is the average of three independent experiments. Errors are the SD of the mean.

(g-h) Equilibrium and kinetic analyses for the N-C interface. **(g)** Waterfall plots of FRET efficiency versus denaturant concentration. **(h)** A kinetic analysis for N-C interface refolding was assayed from the smFRET experiments. Refolding was monitored via an increase in the fraction of bursts with a low acceptor lifetime (**Fig. S4a**, **Table S7**, **Supplementary Note 3**). The curve is the average of three independent experiments. Errors are the SD of the mean.

Fig. S4

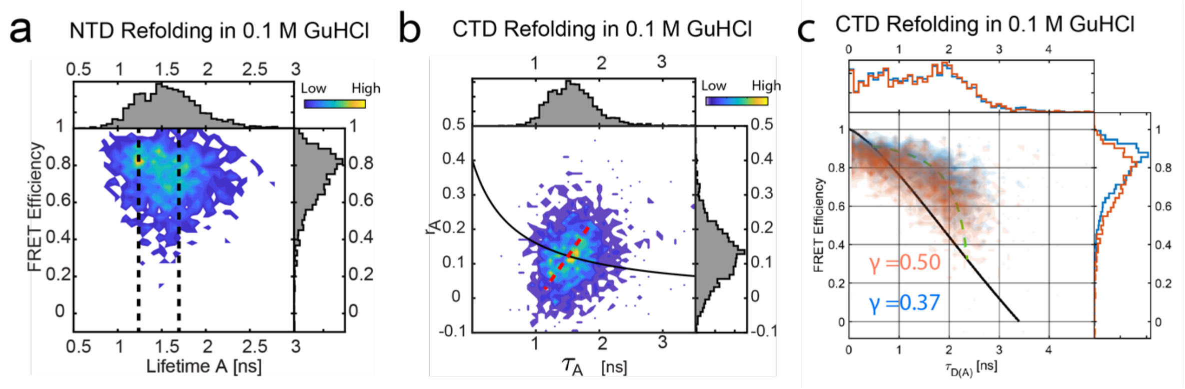

Fig. S4. Quenching of Alexa647 observed during the MBP refolding studies

(a) A 2D-plot of FRET efficiency versus acceptor lifetime (τ_A) during refolding of the CTD. A decrease in the fluorescence lifetime of Alexa647 from 1.7 ns (for the intermediate population) to 1.2 ns is visible upon folding.

(b) A 2D-plot of the steady state anisotropy, r, versus acceptor lifetime (τ_A) for the same refolding measurement as in panel (a). The Perrin equation was fit to the steady state anisotropy vs acceptor lifetime (black line) data to determine the rotational correlation time, ρ. Overall, a ρ of 0.67 ns was found for the acceptor but a clear trend towards a higher steady state anisotropy with increasing lifetime is observable (dashed red line). Note that the steady state anisotropies are below 0.2.

(c) A 2D-plot of FRET efficiency versus $\tau_{D(A)}$ for CTD refolding. Due to the different acceptor lifetimes observable in the measurement, different detection correction factors need to be used. The detection correction factor, γ, was calculated for all the bursts as 0.5 for the unquenched acceptor lifetime of 1.7 ns (shown in orange). This leads the acceptor quenched refolded population of ~0.8 FRET efficiency falling below the static-FRET line (black). When corrected to account for the quenched acceptor lifetime with the corrected γ of 0.37 (shown in blue), the refolded population (now at 0.85 FRET efficiency) correctly falls on the static-FRET line. The dynamic FRET-line is shown as a dashed green curved line.

Fig. S5

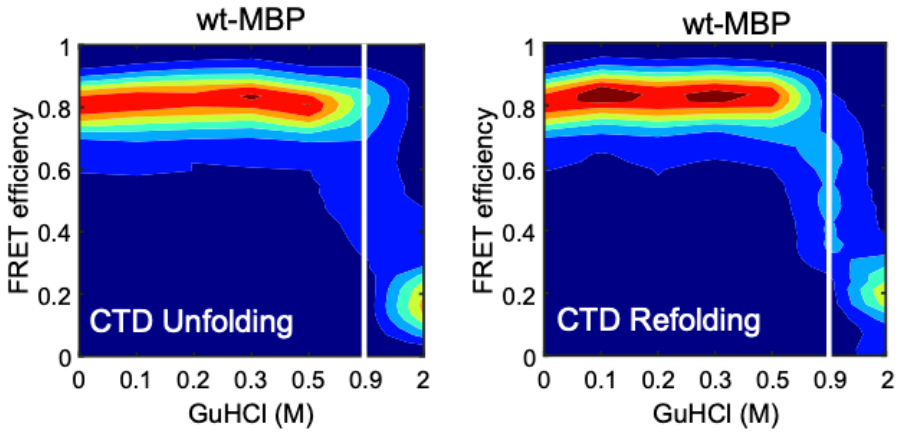

Fig. S5. Equilibrium unfolding-refolding curves of WT-MBP

Waterfall plots of FRET efficiency versus denaturant concentration are presented for WT-MBP during unfolding (left) and refolding (right). Contrary to the refolding traces of the NTD, CTD, and the N-C interface for DM-MBP (**Fig. 1** and **Fig. 2**), an intermediate population is not clearly visible for WT-MBP refolding below 0.9 GuHCl. A white line separates the measurements below and above 0.9 M GuHCl concentration, a concentration below which the dynamic intermediate population is significantly populated during DM-MBP refolding.

Fig. S6

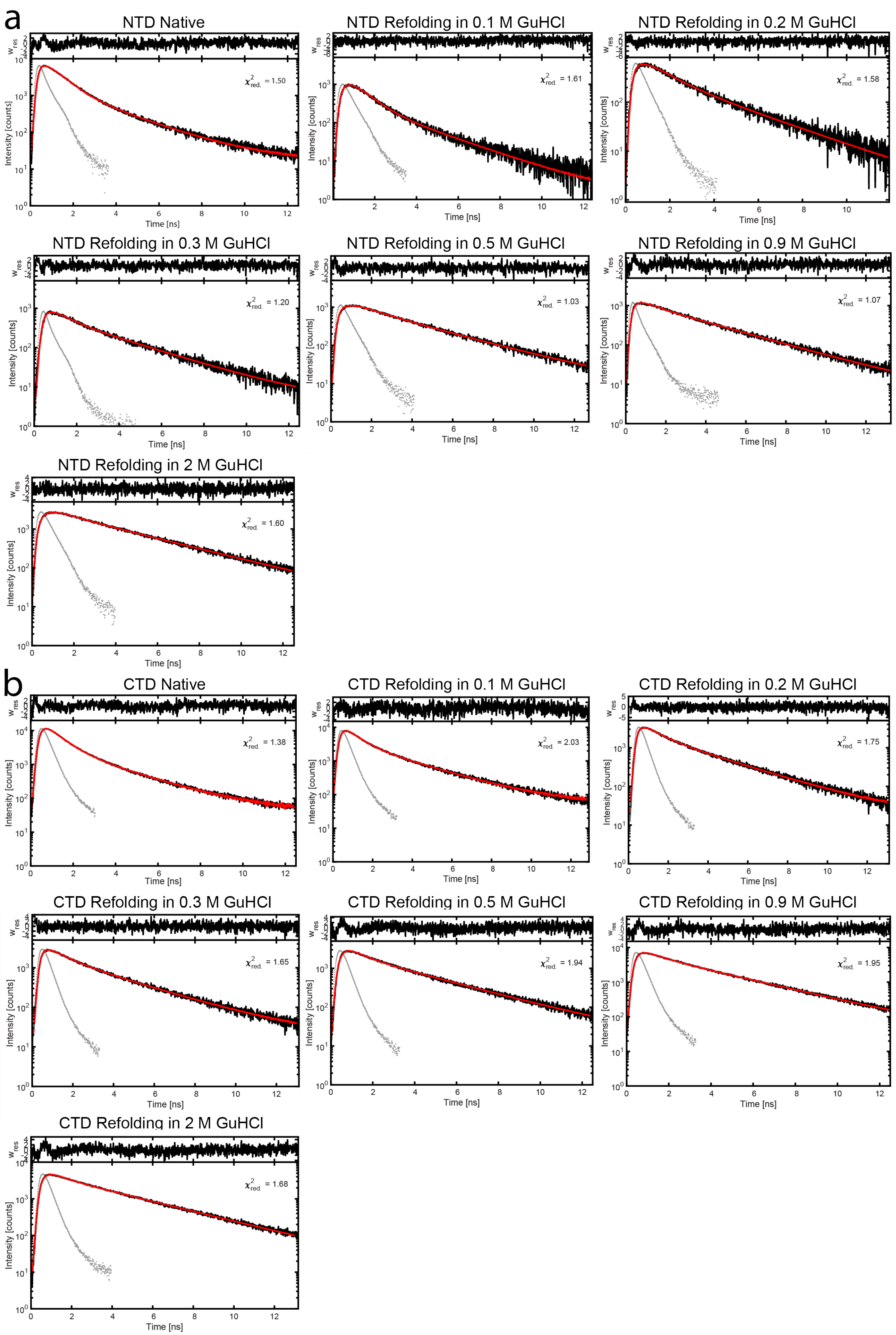
a
NTD Native
$\chi^2_{red.}$ = 1.50
NTD Refolding in 0.1 M GuHCl
$\chi^2_{red.}$ = 1.61
NTD Refolding in 0.2 M GuHCl
$\chi^2_{red.}$ = 1.58
NTD Refolding in 0.3 M GuHCl
$\chi^2_{red.}$ = 1.20
NTD Refolding in 0.5 M GuHCl
$\chi^2_{red.}$ = 1.03
NTD Refolding in 0.9 M GuHCl
$\chi^2_{red.}$ = 1.07
NTD Refolding in 2 M GuHCl
$\chi^2_{red.}$ = 1.60
b
CTD Native
$\chi^2_{red.}$ = 1.38
CTD Refolding in 0.1 M GuHCl
$\chi^2_{red.}$ = 2.03
CTD Refolding in 0.2 M GuHCl
$\chi^2_{red.}$ = 1.75
CTD Refolding in 0.3 M GuHCl
$\chi^2_{red.}$ = 1.65
CTD Refolding in 0.5 M GuHCl
$\chi^2_{red.}$ = 1.94
CTD Refolding in 0.9 M GuHCl
$\chi^2_{red.}$ = 1.95
CTD Refolding in 2 M GuHCl
$\chi^2_{red.}$ = 1.68
w_{res}
Intensity [counts]
Time [ns]

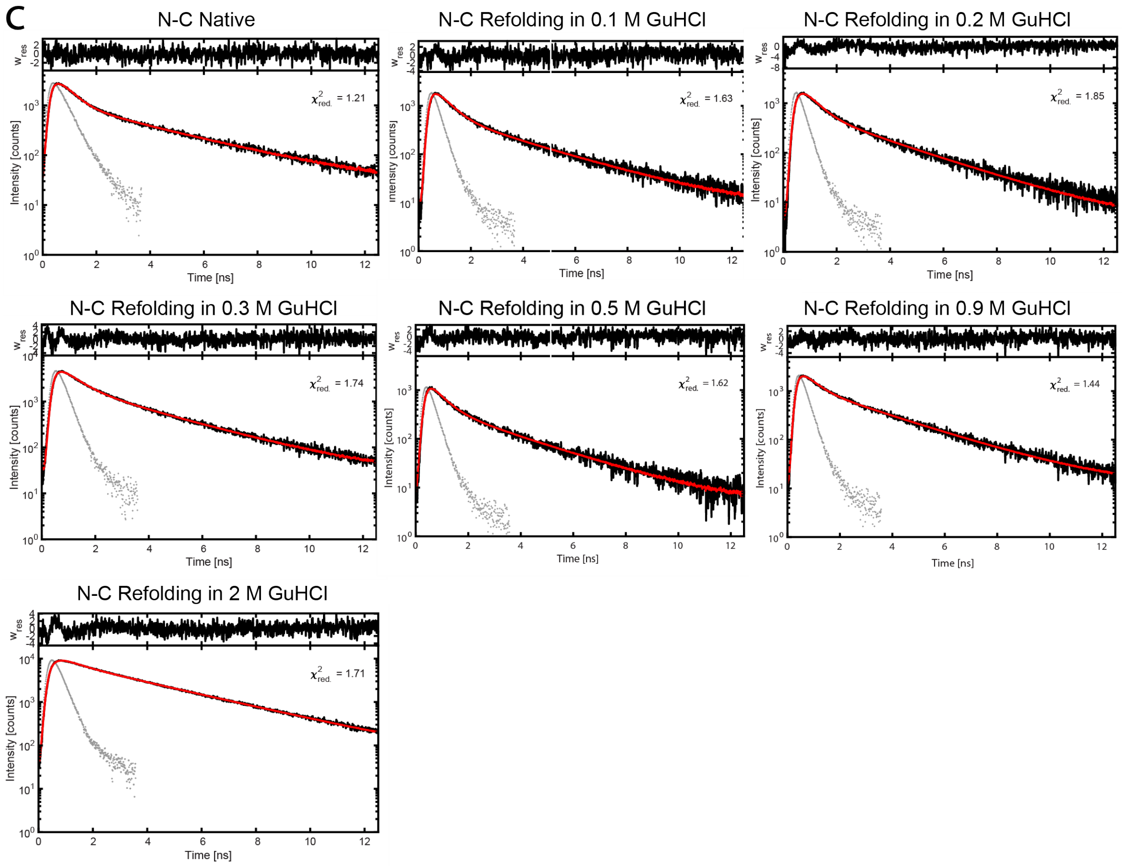

Fig. S6. Analysis of the donor fluorescence lifetime for double-labeled molecules of DM-MBP from 2C FRET measurements on the NTD, CTD and the N-C interface.

(a-c) To extract the donor lifetimes defining the conformational states present during DM-MBP refolding, the donor fluorescence decay (black) was determined by summing all photons from the selected double-labeled bursts and fitted with a bi-exponential function (red line) for the NTD **(a)**, CTD **(b)** and the N-C interface **(c)**. All three constructs were labeled with Atto532 and Alexa647. The lifetime fit was performed on the intensity decay by a convolution with the instrument response function (grey). The quality of the fit model was judged by the χ^2_{red} value. The upper panel shows the weighted residuals from the fit. The donor lifetime values obtained from the fits are summarized in **Table S4** and plotted as white squares in **Fig. 2a**. These values were employed to carry out a FRET efficiency versus donor lifetime analysis as well as to analyze the conformational dynamics using the dynamic photon distribution analysis.

Fig. S7

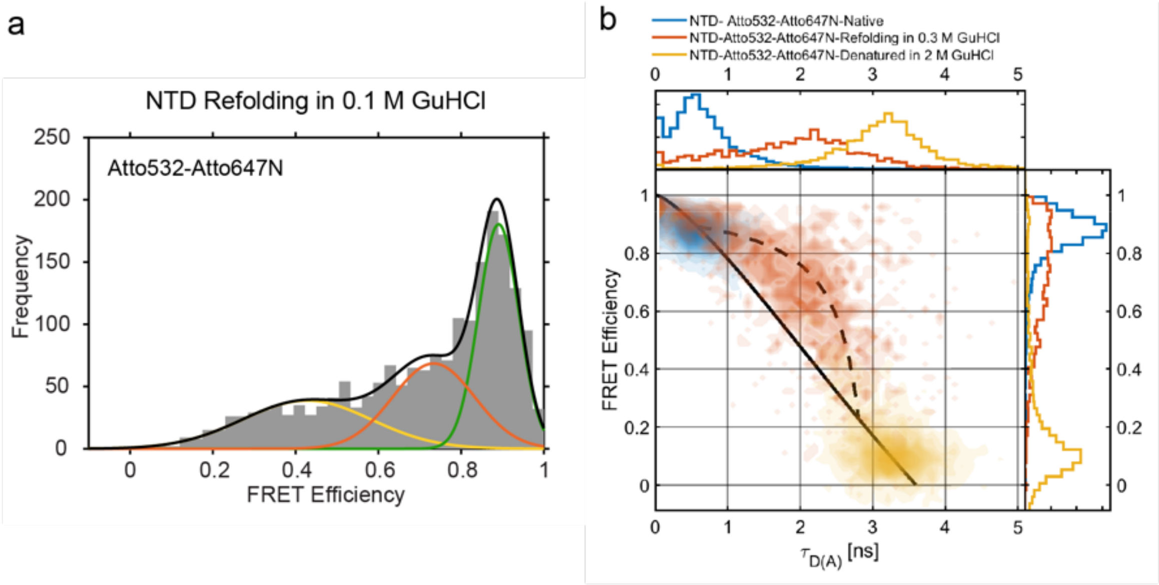

Fig. S7. Conservation of conformational dynamics using a different acceptor in DM-MBP refolding measurements.

(a) A smFRET histogram of Atto532-Atto647N labeled NTD during the first 3000 s of refolding. The intermediate population found when using Alexa647 was still preserved when using Atto647N as an alternate acceptor.

(b) A 2D-plot of FRET efficiency versus donor lifetime in the presence of an acceptor ($\tau_{D(A)}$) (*E*-*τ* plot) for the NTD construct labeled with Atto532 and Atto647N. The FRET histograms for the native conformation (blue), refolding in 0.3 M GuHCl (orange) and in the denatured state (i.e. in 2.0 M GuHCl) (yellow) are shown. Note that the refolding NTD has a similar intermediate population possessing sub-millisecond dynamics as observed when using Alexa647 as an acceptor (Fig. 2b). These results suggest that the dynamics originate from the protein are not dependent on the acceptor used.

Fig. S8

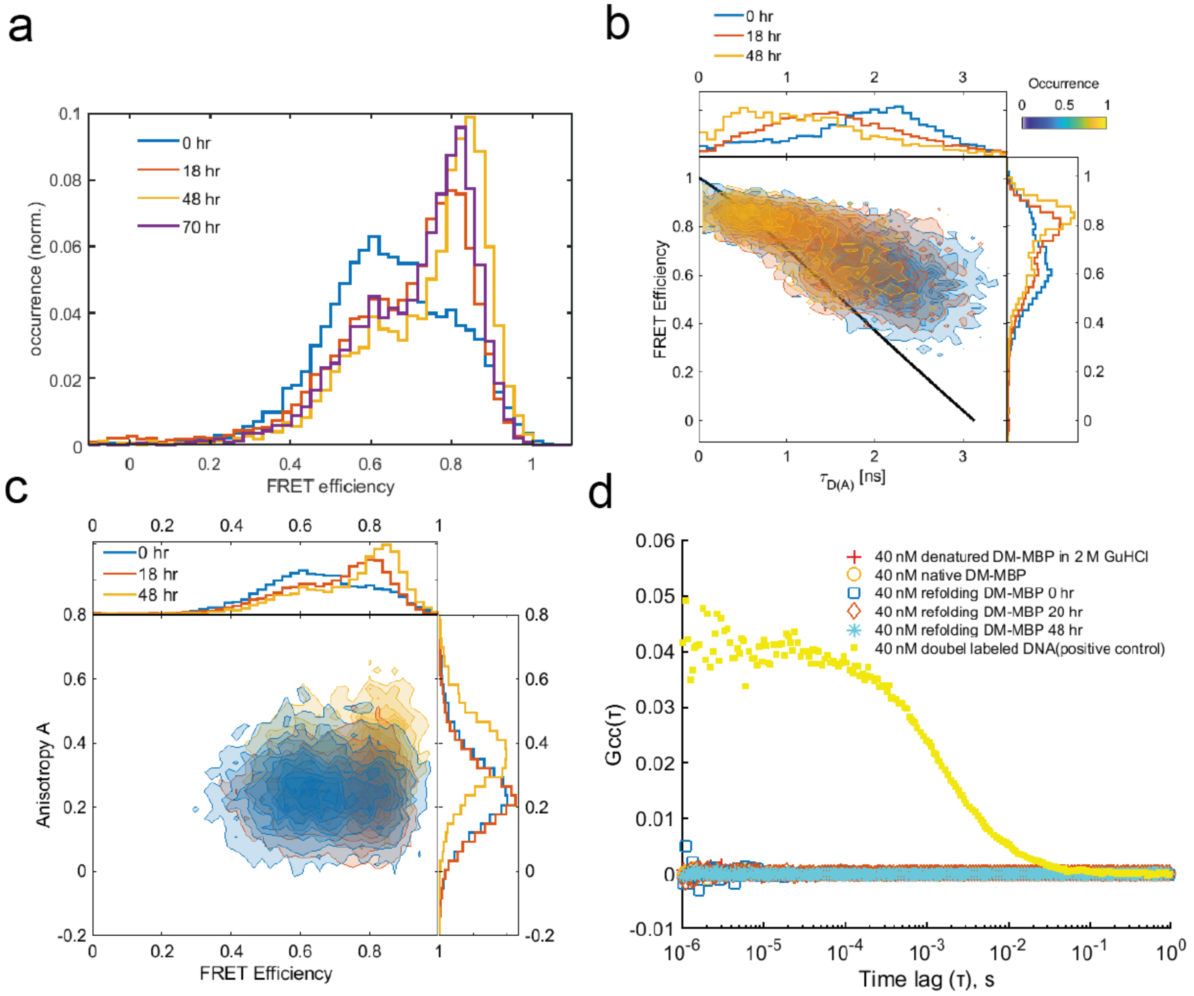

Fig. S8: Following the refolding of DM-MBP in 0.3 M GuHCl over 3 days

(a-c) DM-MBP labeled with Atto532 and Alexa647 was denatured in 3 M GuHCl and diluted to 40 nM concentration to start the refolding reaction. Small volumes from the same reaction mixture were taken and further diluted to picomolar concentrations for the smFRET experiments at the mentioned time points.

(a) SmFRET efficiency histograms of the refolding of the NTD construct of DM-MBP measured in 0.3 M GuHCl at 0, 18, 48 and 70 hr from the start of the reaction are shown.

(b-c) 2D plot of FRET efficiency versus donor fluorescence lifetime **(b)** and 2D plot of acceptor anisotropy versus FRET efficiency **(c)** are shown for the same smFRET measurements plotted in **(a)**.

(d) FCCS experiments performed to check for aggregation during the measurements mentioned in **(a).** The measurements were performed at 40 nM total protein concentration. For clarity, the 70 hr measurement is not included in **(b-d)** as the results are similar to the 48 hr measurement.

Fig. S9

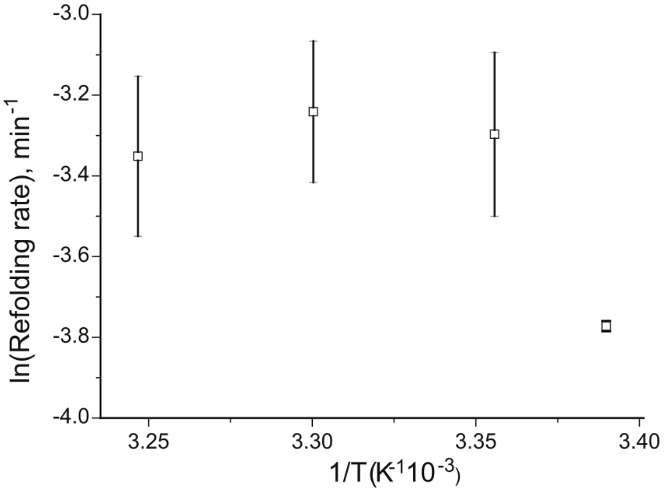

Fig. S9. Arrhenius plot for the refolding rates of the NTD construct at different temperatures measured by smFRET.

SmFRET refolding measurements of the NTD construct were performed in 0.1 M GuHCl at temperatures of 22 °C, 25 °C, 30 °C and 35 °C. The rates are plotted as a function of temperature in an Arrhenius plot. The error bars are the standard deviations from three independent experiments.

Fig. S10

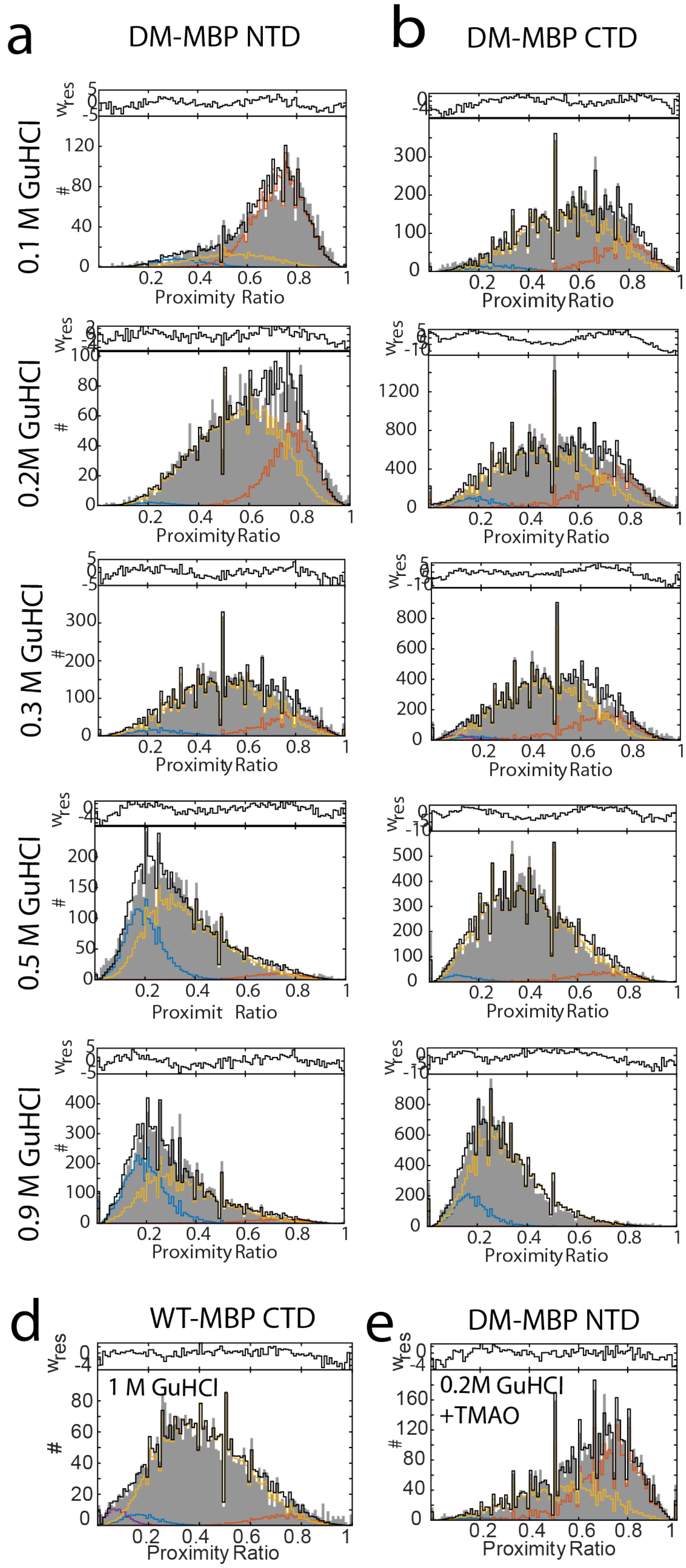

a
DM-MBP NTD
b
DM-MBP CTD
c
DM-MBP N-C
0.1 M GuHCl
0.2M GuHCl
0.3 M GuHCl
0.5 M GuHCl
0.9 M GuHCl
Proximity Ratio
Proximit Ratio
d
WT-MBP CTD
1 M GuHCl
e
DM-MBP NTD
0.2M GuHCl
+TMAO

Fig. S10. Conformational dynamics quantified using the dynamic photon distribution analysis

(a-c) The results of a dynamic PDA analysis for the NTD (a), CTD (b), and for the N-C interface (c) constructs of DM-MBP labeled with Atto532 and Alexa647 are shown. Representative single molecule proximity FRET histograms using a binning of 1 ms are plotted. The analyzed refolding measurements were performed in 0.1, 0.2, 0.3, 0.5, and 0.9 M GuHCl concentrations. For the 0.1 m GuHCl refolding measurements, only the initial 2000 s were analyzed as refolding occurs over ca 30 minutes. For all other GuHCl concentrations, burst during the entire measurement of 10,800 - 18,000 s were analyzed.

(d) A dynamic PDA analysis was performed for the CTD construct of WT-MBP in 1 M GuHCl. As above, the protein was labeled with Atto532 and Alexa647.

(e) A dynamic PDA analysis of the NTD construct of DM-MBP labeled with Atto532 and Alexa647 refolding in 0.2 M GuHCl supplemented with 500 mM TMAO.

For all panels, the proximity ratio histograms are shown in grey. The dynamic PDA fitting is highlighted with a black outline in the histogram. In dynamic PDA fitting, the refolded population is highlighted in red, the unfolded state in blue, and yellow represents the contribution of the interconverting species between the folded and unfolded states to the histogram. A global fit was performed using smFRET proximity histograms for 0.5, 1 and 1.5 ms bins to quantify the transition rates (**Table S6**). Only the 1 ms binned histograms are shown here for clarity.

Fig. S11

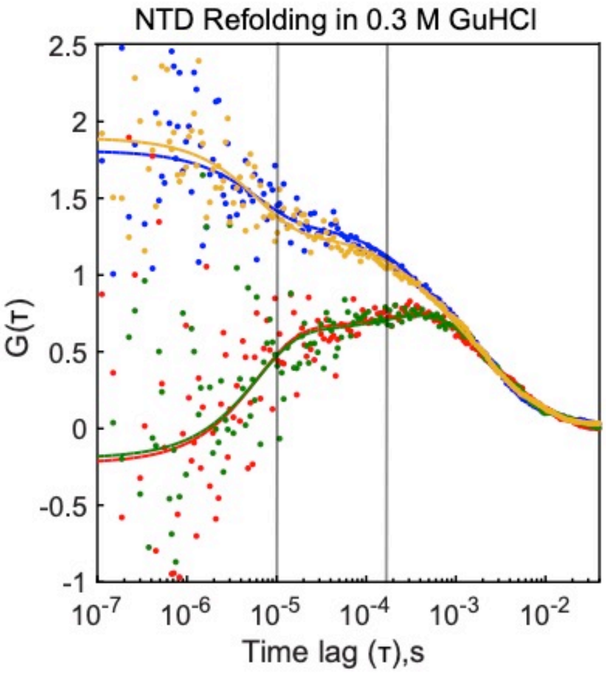

Fig. S11. Exemplary global filtered FCS analysis for the NTD refolding in 0.3 M GuHCl. The two auto correlation functions for sub-population 1 (yellow) and sub-population 2 (blue) and two cross correlation functions for sub-population 1 x sub-population 2 (red) and for sub-population 2 x sub-population 1 (green) are shown for DM-MBP NTD refolding in 0.3 M GuHCl. A global analysis of all the four correlation functions was performed over the GuHCl concentrations (0.3, 0.5 and 0.9 M GuHCl) to obtain a robust fit. The highlighted two relaxation times are 9.7 μs and 175 μs.

Fig. S12

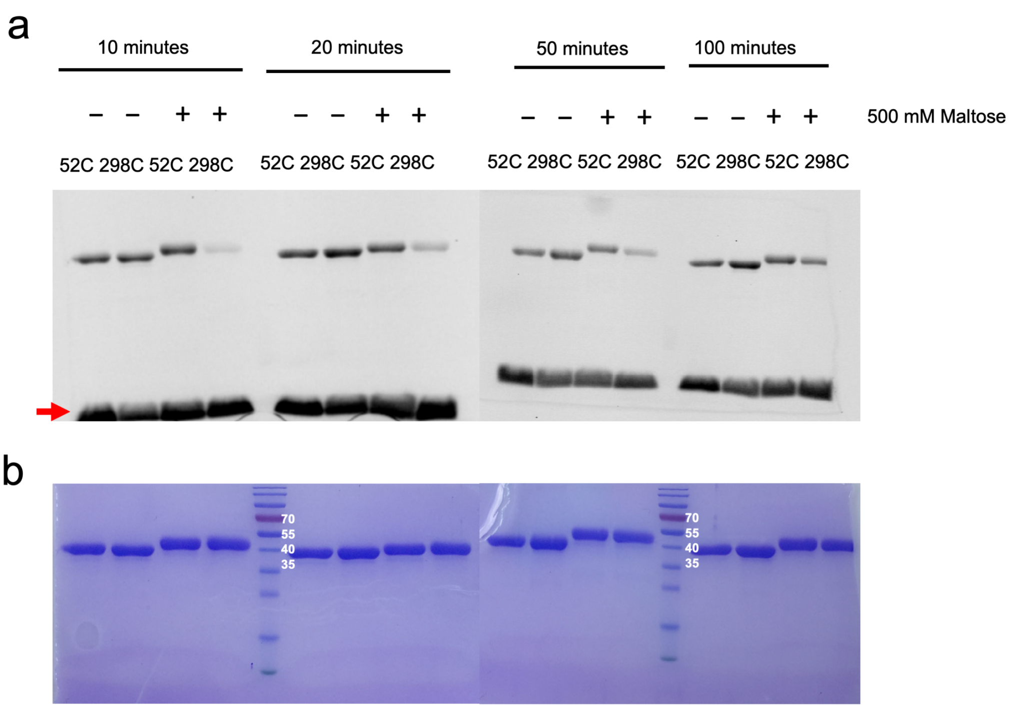

Fig. S12. The P298C position is inaccessible for cysteine-maleimide labeling when maltose is bound to DM-MBP.

(a) Labeling of Atto565-maleimide conjugate to single cysteine mutants A52C and P298C was monitored after 10 min, 20 min, 50 min and 100 minutes from the start of the reaction. Labeling was performed in the absence and the presence of 500 mM maltose for the two mutants. Afterwards, the sample was loaded and run on an SDS-PAGE gel. The signal of the Atto565 fluorescence was collected by excitation with UV-light. Maltose binding to DM-MBP reduces the accessibility of position P298C and thereby decreasing the amount of labeling, even after 50 minutes. In contrast, labeling to position A52C was evident even within 10 minutes. A52C serves as a positive control for the labeling reaction at a solvent accessible position in the presence of maltose. It also helps to rule out any viscosity effects arising from the dissolve 500 mM maltose on the labeling kinetics. Free unlabeled dye is indicated with a red arrow on the scanned gel. The presence of 500 mM maltose in the reaction slows down the migration of the SDS-denatured protein on the gel.

(b) A coomassie staining of the same SDS-PAGE gels after scanning for Atto565 emission. The molecular weights of the marker proteins are given in kDa. The amount of the protein loaded for all reactions was equal.

Fig. S13

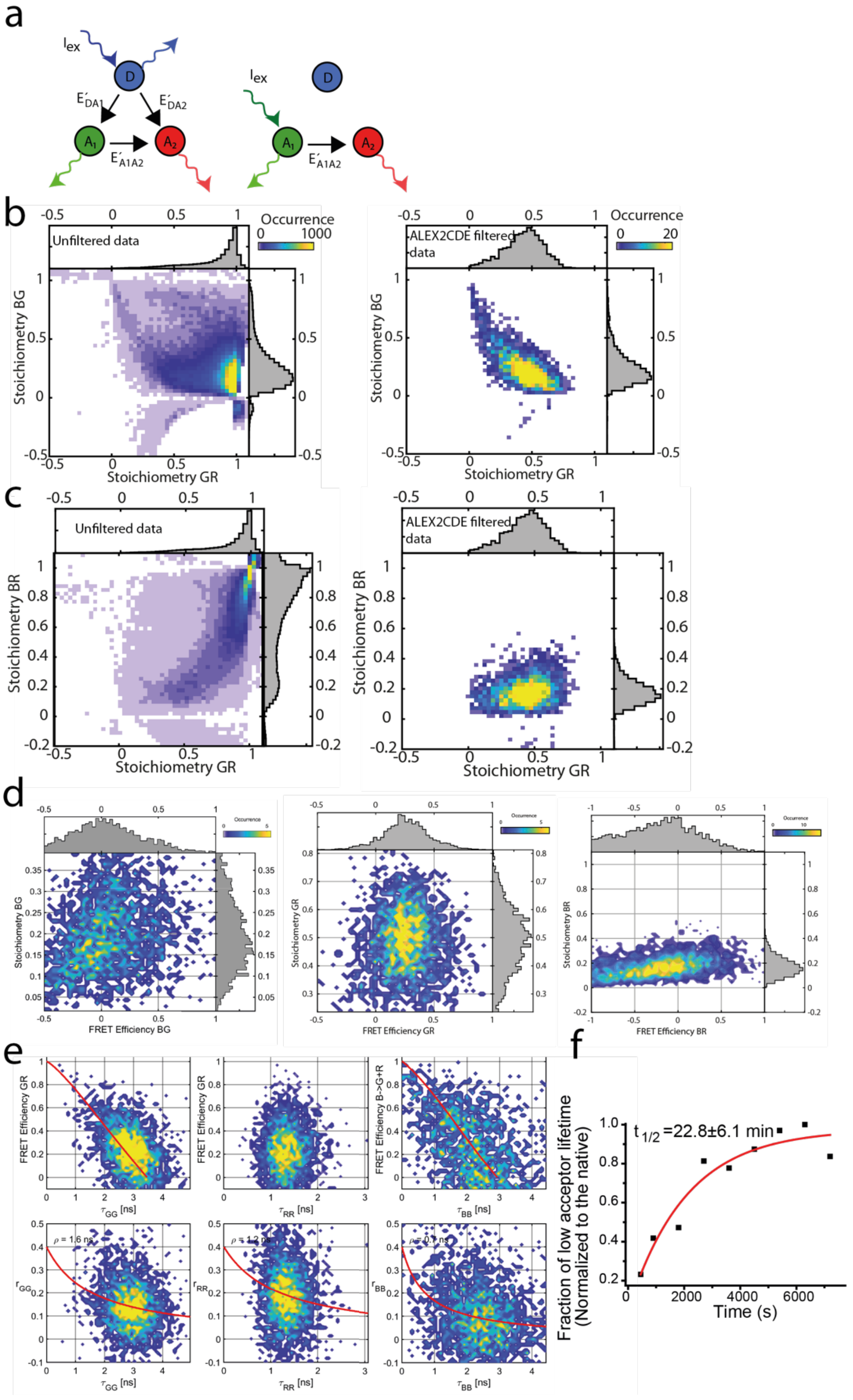
a
b
Unfiltered data
Occurrence
ALEX2CDE filtered data
Stoichiometry BG
Stoichiometry GR
c
Stoichiometry BR
d
FRET Efficiency BG
FRET Efficiency GR
FRET Efficiency BR
e
FRET Efficiency GR
FRET Efficiency B->G+R
f
$t_{1/2}$=22.8±6.1 min
Fraction of low acceptor lifetime
(Normalized to the native)
Time (s)

Fig. S13. 3C smFRET analysis of DM-MBP using MFD-PIE

(a) A schematic of 3C smFRET. In the left panel, the blue fluorophore is excited (donor, D) and can transfer energy to the green dye (first acceptor, A_1) or to the red dye (second acceptor, A_2). Furthermore, an excited green dye can transfer its energy to the red dye. The rate of energy transfer between the green and red dyes can be determined by directly exciting the green fluorophore with a green laser pulse, as shown in the right panel.

(b) BG-GR selection criterion used for analyzing three-dye labeled molecules. Left panel: A 2D plot of the BG stoichiometry versus GR stoichiometry for all the bursts. Right panel: The same 2D stoichiometry plot after applying the ALEX 2C 2CDE filter for all three FRET pair combinations.

(c) BR-GR selection criterion used for analyzing three-dye labeled molecules. Note that the selected molecules for further 3C FRET analysis must pass both the BG-GR and BR-GR selection criterion.

(d) Representative 3C FRET data of 3 M GuHCl denatured triple-labeled DM-MBP. Triple-labeled molecules were selected that passed the criteria discussed in panels B and C**.** Left panel: The stoichiometry BG versus FRET efficiency BG is plotted. Middle panel: The stoichiometry GR versus FRET efficiency GR is plotted. Right panel: The stoichiometry BR versus FRET efficiency BR is plotted.

(e) Burst-wise lifetime and anisotropy plots of 3C FRET data for 3 M GuHCl denatured triple-labeled DM-MBP. Upper panels, from left to right: The FRET efficiency GR versus green dye lifetime (τ_{GG}), FRET efficiency GR versus red dye lifetime (τ_{RR}) and FRET efficiency B→G+R versus blue dye lifetime (τ_{BB}). Red lines represent the ideal relationship between FRET efficiency and donor (green/blue) lifetime for static FRET states. Lower panel, from left to right: The burst-wise anisotropy values for donor (blue and green) and acceptor (red) fluorophores versus their respective lifetimes are plotted. The red lines are the fits to the Perrin equation.

(f) A kinetic analysis of DM-MBP refolding of the 3C smFRET data assayed as explained in **Fig. S4** for the CTD construct measured with 2C smFRET (**Table S1** and **Table S8**).

Fig. S14

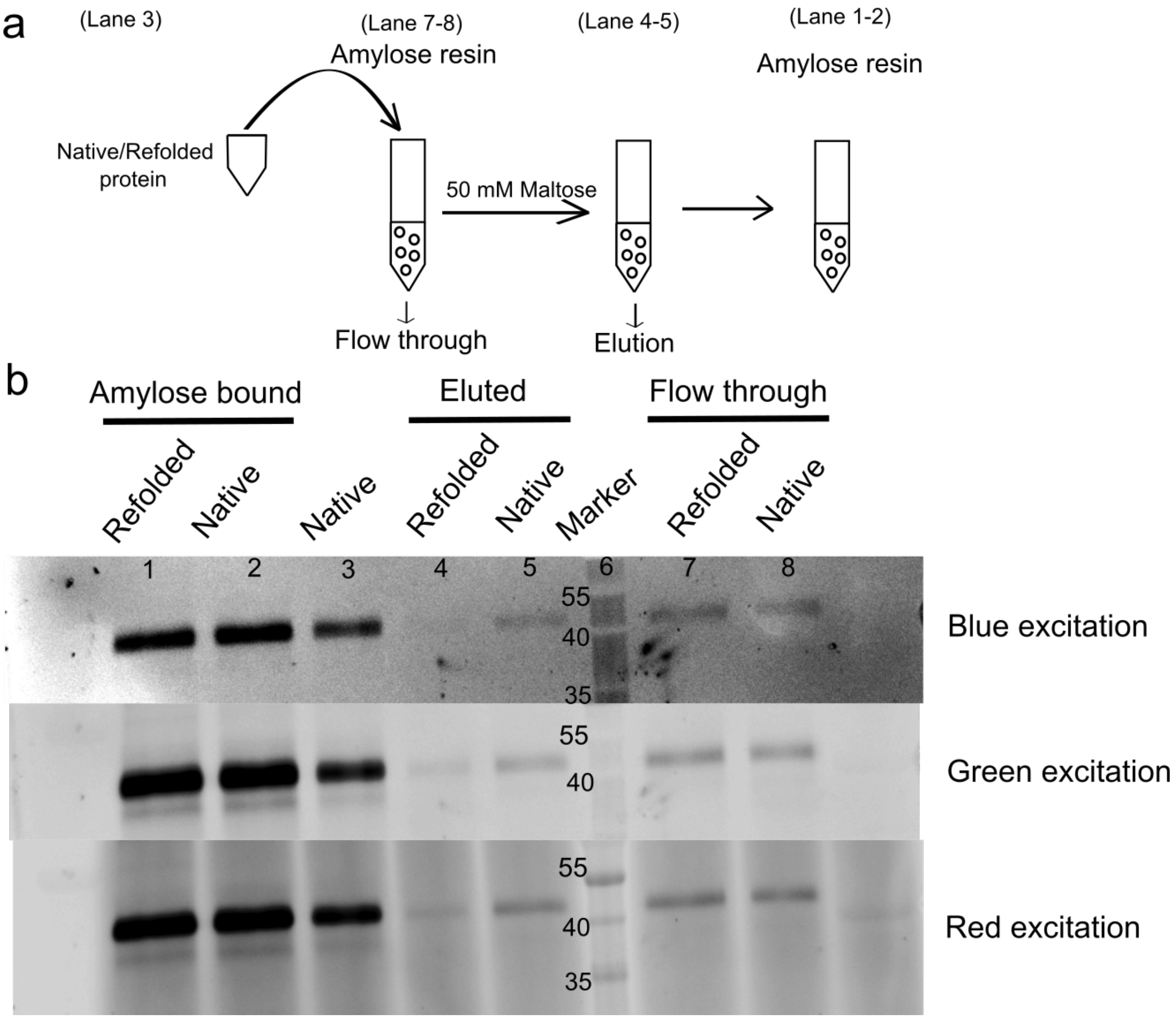

Fig. S14 Functional assay of refolded DM-MBP labeled with Atto488, Atto565, and Alexa647

(a) A schematic showing the different fractions collected in the experiment. Maltose is the natural ligand for MBP. Amylose resins are generally used to immobilize and purify maltose binding proteins.

(b) An SDS-PAGE gel of native and refolded protein run over an amylose column. The initial solution of native protein is shown in lane 3. A comparison is shown between refolded and natively folded triple-labeled DM-MBP that was eluted from the column with 50 mM maltose (lanes 4-5) as well as what flowed through the column (lanes (7-8). After elution, we also denatured the protein still bound to the column (lanes 1-2). The protein marker (lane 6) shows the mentioned molecular weights in kDa. Under both conditions, triple-labeled DM-MBP was able to bind to the

amylose column. The assay shows the ability of refolded triple-labeled DM-MBP to bind to amylose and to be displaced by maltose binding with efficiencies similarity to that of the three-color-labeled native protein. Hence, the three-color labeled protein still refolds. For refolding, triple-color DM-MBP was first denatured in 3 M GuHCl and allowed to refold for 2 hr upon dilution to 0.1 GuHCl concentration. The differing amount of protein detected during flow-through and elution results from dilution effects. The different fluorophores attached to DM-MBP proteins were detected with blue (480 nm, upper row), green (560 nm, middle row) and red (640 nm, lower row) excitation respectively.

Fig. S15

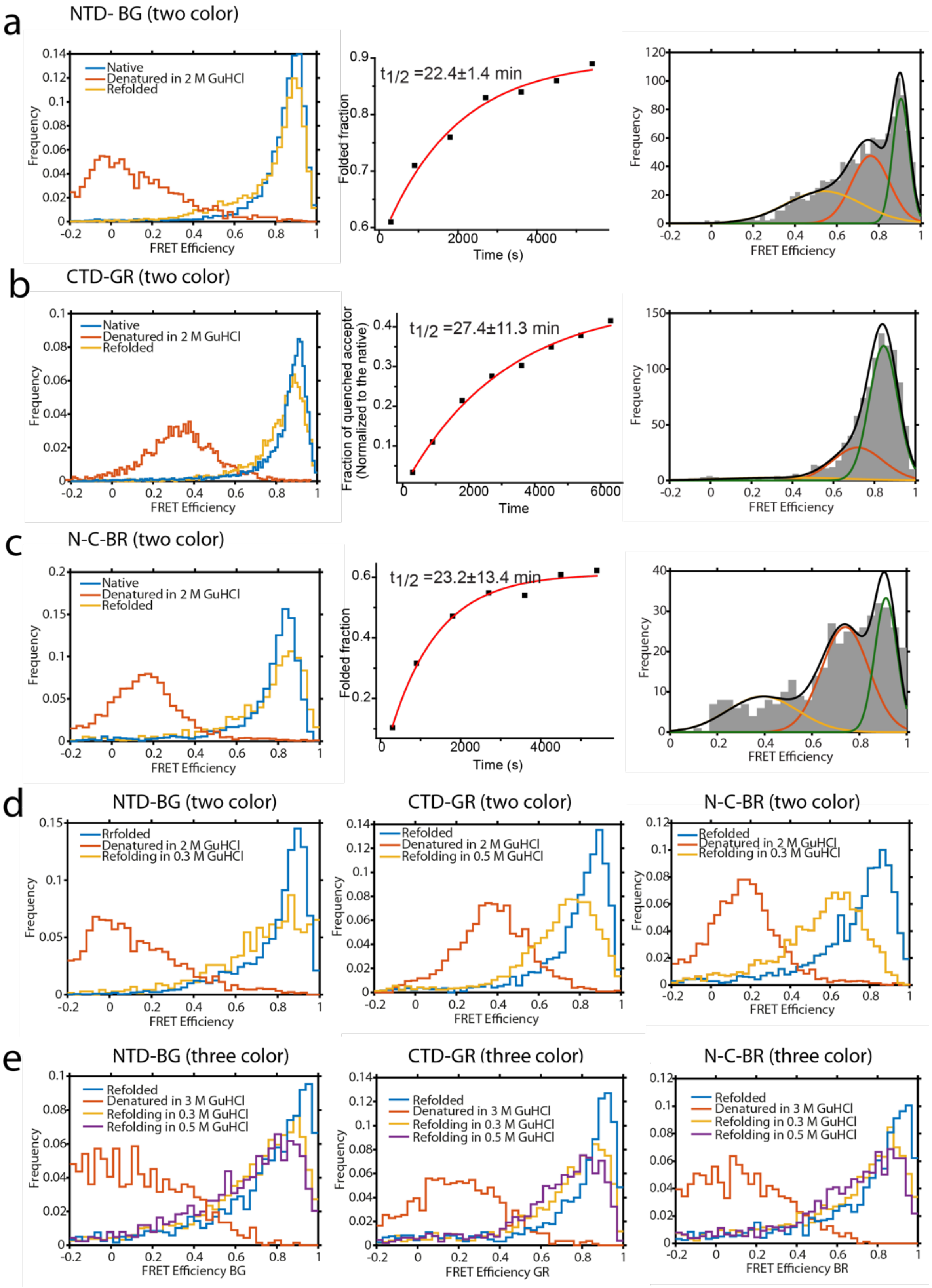
a
NTD- BG (two color)
Native
Denatured in 2 M GuHCl
Refolded
Frequency
FRET Efficiency
$t_{1/2}$ =22.4±1.4 min
Folded fraction
Time (s)
Frequency
FRET Efficiency
b
CTD-GR (two color)
Native
Denatured in 2 M GuHCl
Refolded
Frequency
FRET Efficiency
$t_{1/2}$ =27.4±11.3 min
Fraction of quenched acceptor (Normalized to the native)
Time
Frequency
FRET Efficiency
c
N-C-BR (two color)
Native
Denatured in 2 M GuHCl
Refolded
Frequency
FRET Efficiency
$t_{1/2}$ =23.2±13.4 min
Folded fraction
Time (s)
Frequency
FRET Efficiency
d
NTD-BG (two color)
Rrfolded
Denatured in 2 M GuHCl
Refolding in 0.3 M GuHCl
Frequency
FRET Efficiency
CTD-GR (two color)
Refolded
Denatured in 2 M GuHCl
Refolding in 0.5 M GuHCl
Frequency
FRET Efficiency
N-C-BR (two color)
Refolded
Denatured in 2 M GuHCl
Refolding in 0.3 M GuHCl
Frequency
FRET Efficiency
e
NTD-BG (three color)
Refolded
Denatured in 3 M GuHCl
Refolding in 0.3 M GuHCl
Refolding in 0.5 M GuHCl
Frequency
FRET Efficiency BG
CTD-GR (three color)
Refolded
Denatured in 3 M GuHCl
Refolding in 0.3 M GuHCl
Refolding in 0.5 M GuHCl
Frequency
FRET Efficiency GR
N-C-BR (three color)
Refolded
Denatured in 3 M GuHCl
Refolding in 0.3 M GuHCl
Refolding in 0.5 M GuHCl
Frequency
FRET Efficiency BR

Fig. S15. 2C smFRET control measurements for the 3C smFRET measurements

(a) 2C BG-FRET control measurements of the NTD. The NTD was labeled with Atto488 and Atto565 as used for 3C smFRET. Left panel: 2C smFRET histograms comparing the native (blue), 2 M GuHCl denatured (orange) and refolded NTD (yellow) conformations. Middle panel: A kinetic analysis of the smFRET histogram during refolding of the NTD (**Table S1**). Right panel: SmFRET histogram for the entire refolding measurement shown in the middle panel. The unfolded state is shown in green, the intermediate population in yellow and the refolded state in orange.

(b) 2C GR-FRET control measurements of the CTD. The CTD was labeled with Atto565 and Alexa647 as used for 3C smFRET.

(c) 2C BR-FRET control measurements of the N-C interface. The N-C interface was labeled with Atto488 and Alexa647 as used for 3C smFRET.

(d-e) Comparison of the 1-D smFRET histograms for 2C smFRET control measurements (d) and for the 3C smFRET experiments (e). *Left panels*: Left panels: BG FRET histograms of the NTD were compared under the conditions of refolded (in 0.1 GuHCl, blue), 2 M GuHCl denatured (orange), and refolding in 0.3 M GuHCl (yellow) and 0.5 M GuHCl (purple). The 2C FRET NTD was labeled with same fluorophores (i.e. Atto488 and Atto565) as used for the 3C smFRET measurements. *Middle panels*: GR FRET histograms of the CTD were compared under the conditions of refolded (in 0.1 GuHCl, blue), 2 M GuHCl denatured (orange), and refolding in 0.3 M GuHCl (yellow) and 0.5 M GuHCl (purple). The 2C FRET CTD was labeled with same fluorophores (i.e. Atto565 and Alexa647) as used for the 3C smFRET measurements. *Right panels*: BR FRET histograms of N-C interface were compared under the conditions of refolded (in 0.1 GuHCl, blue), 2 M GuHCl denatured (orange), and refolding in 0.3 M GuHCl (yellow) and 0.5 M GuHCl (purple). The 2C FRET N-C interface was labeled with same fluorophores (i.e. Atto488 and Alexa647) as used for the 3C smFRET measurements.

Fig. S16

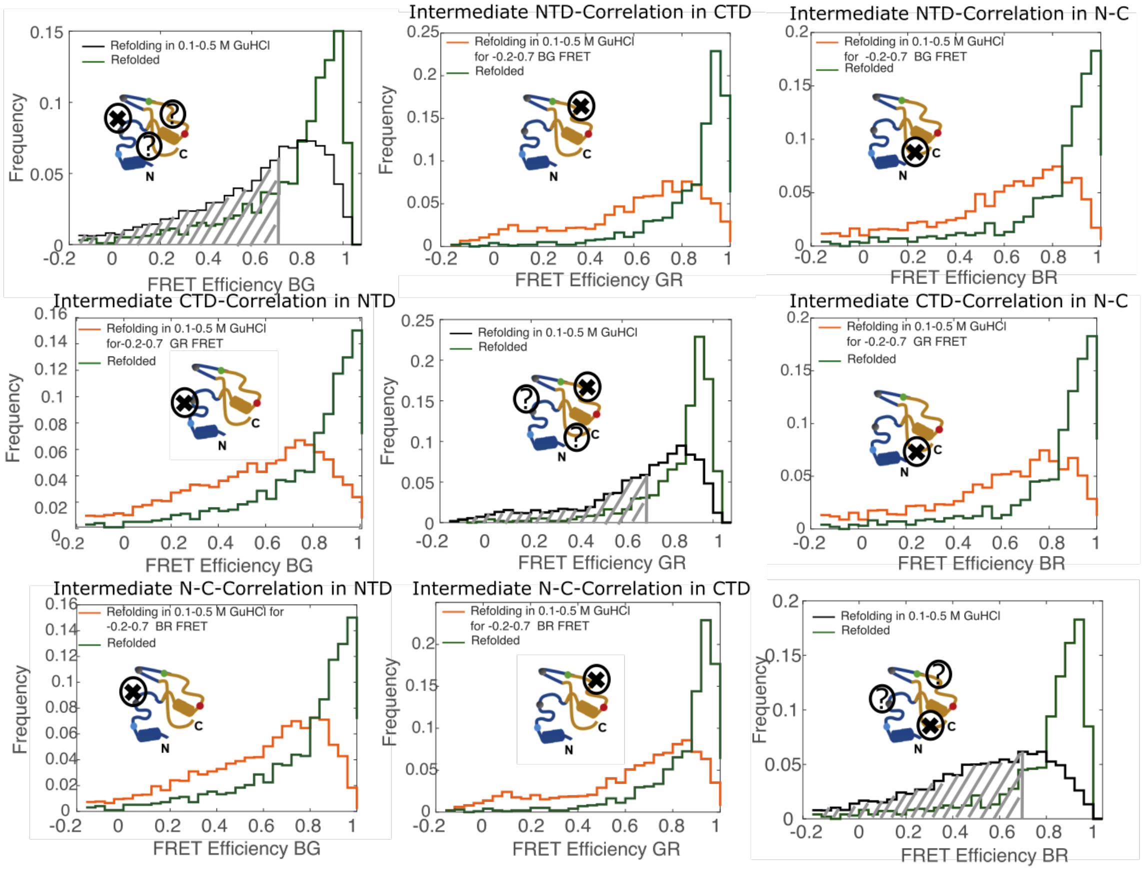

Fig. S16. Correlations found by 3C FRET in folded and an intermediate population during the folding of DM-MBP

A comparison of the 3C smFRET histograms for molecules in the intermediate population measured in 0.1 - 0.5 M GuHCl concentrations (E < 0.7) for one FRET pair compared to the smFRET histograms of refolded protein for the respective FRET pair in the 3C measurement (green). The smFRET histogram of all molecules measured between 0.1 and 0.5 M GuHCl is shown in black where the molecules selected with E < 0.7 are highlighted in grey. The corresponding smFRET efficiency histograms of the selected molecules for the other two FRET pairs are shown in orange. Histograms for the NTD (GR) are on the left, for the CTD in the middle and for the N-C interface on the right. Molecules selected for folded NTD are shown in the top row, for folded CTD in the middle row and for a native-like N-C interface in the bottom row.

Fig. S17

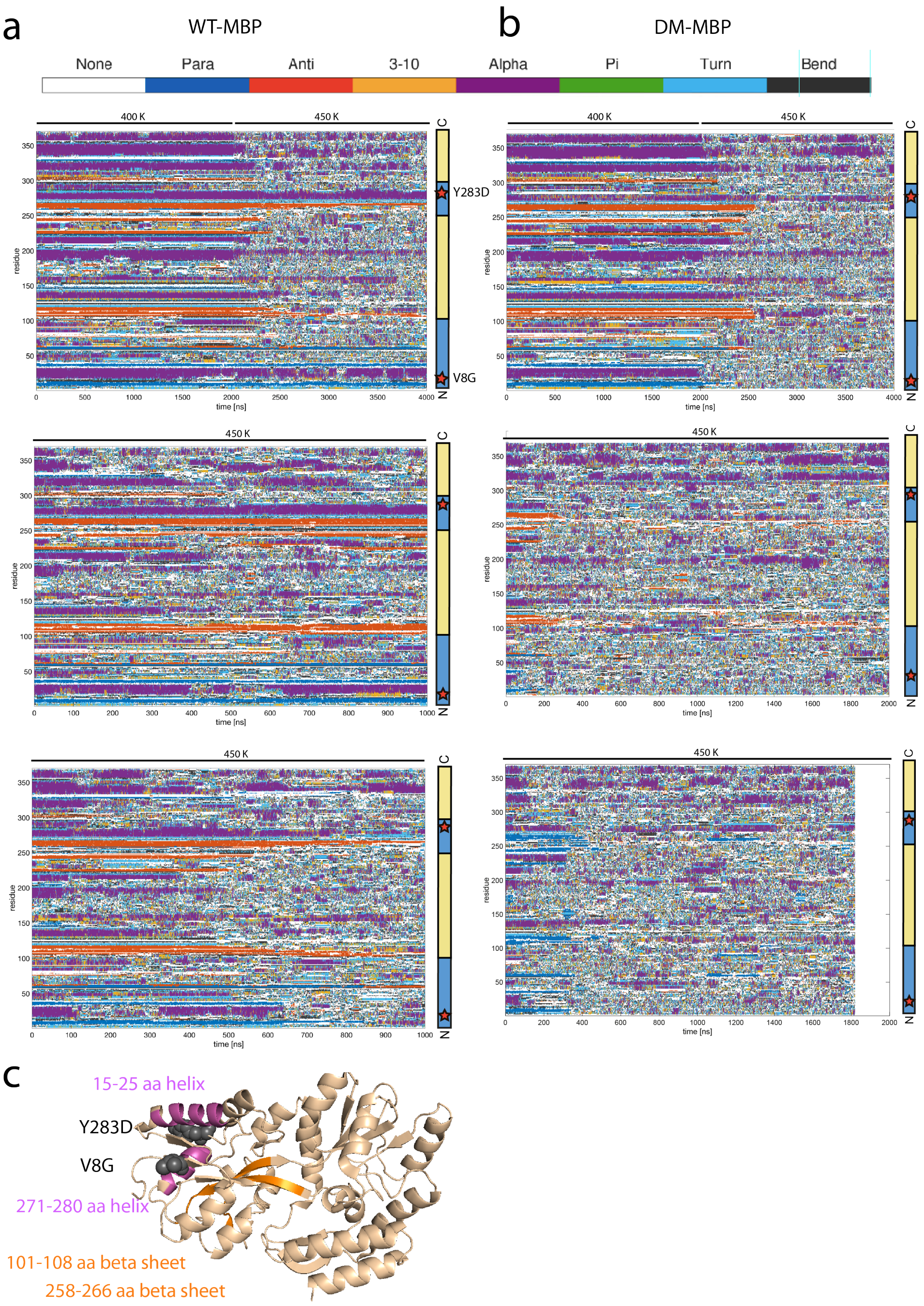

Fig. S17. Repeats of MD simulations performed on WT-MBP and DM-MBP for temperature induced unfolding.

(a-b) DSSP (Definition of Secondary Structure of Proteins) based secondary structure annotation plot for MD simulations based on PDB ID:1OMP were performed on WT-MBP **(a)** and on DM-MBP **(b)** for temperature induced unfolding. The plot shows the various secondary structures

present in amino acid sequence from N to C terminus. *Upper panels*: The simulation was performed at 400 K for the initial 2 *µ*s to reach equilibrium and then temperature was than increased to 450 K for another 2 *µ*s to induce unfolding. *Middle and lower panels*: The repeats of simulation were performed at 450 K for 1-2 *µ*s. Random coil is shown in white, parallel beta sheet in blue, anti-parallel beta sheet in orange, 3-10 helix in yellow, alpha helix in purple, Pi helix in green, beta turn in sky blue and bends in black. The predominantly found alpha helices and anti-parallel beta sheets secondary structures can be seen throughout the MBP structure. Double mutations present in DM-MBP, V8G and Y283D, are depicted in the right panel in MBP the sequence. As significant changes in the MBP structure were not observed during equilibration step as shown in the upper panel, we perform the unfolding repeat/s without first equilibrating the structure.

(c) Secondary structures preserved in the MD simulations performed on WT-MBP after heating at 450 K are highlighted on the MBP structure. Alpha helices are shown in purple, anti-parallel beta sheets are shown in orange. Double mutations in dark grey and three dye labels are shown in their respective color codes used for the three-color smFRET experiments.

Fig. S18

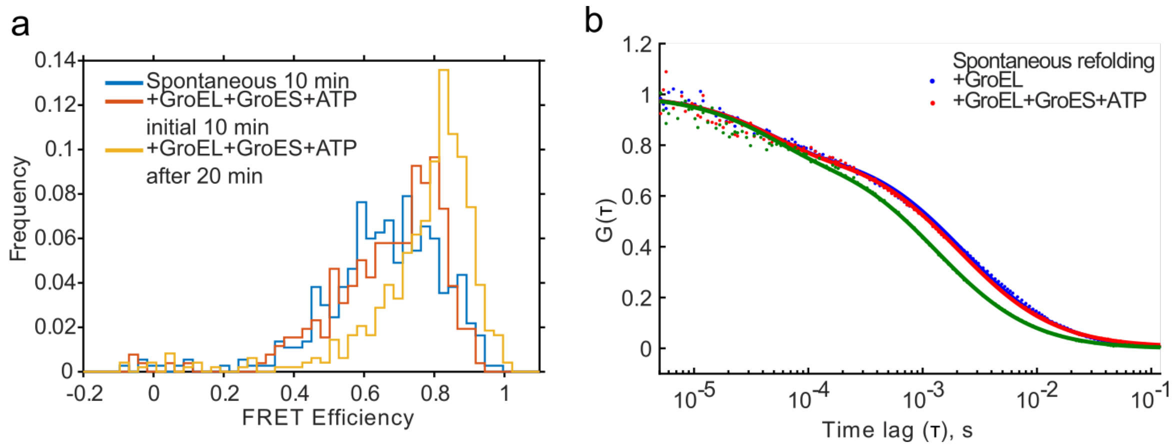

Fig. S18. Characterization of assisted refolding of DM-MBP by the GroEL/ES chaperonins. **(a)** SmFRET efficiency histograms for the NTD construct of spontaneously refolding DM-MBP during the initial 10 min (blue) of refolding, and in the presence of GroEL/ES/ATP during the initial 10 min (orange) and after 20 min (yellow). **(b)** FCS curves for the free spontaneously refolding DM-MBP (green), GroEL bound (blue) and in presence of GroEL/ES/ATP (red).

Supplementary Tables

Table S1

	Refolding rate (min^{-1})	$t_{1/2}$ of refolding (min)
WT-MBP[a]	2.4±0.5	0.29±0.07
DM-MBP[a]	0.028±0.003	24.7±2.7
NTD (52C-298C) Atto532-Alexa647[b] at RT (22° C)	0.023±0.001	22.4±10.0
NTD (52C-298C) Atto532-Alexa647[b] at 25° C	0.030±0.007	18.8±3.4
NTD (52C-298C) Atto532-Alexa647[b] at 30° C	0.039±0.006	17.8±2.8
NTD (52C-298C) Atto532-Alexa647[b] at 35° C	0.036±0.007	19.4±3.8
NTD (52C-298C) Atto488-Atto565[b]	0.030±0.001	22.5±1.4
CTD (175C-298C) Atto532-Alexa647[b]	0.039±0.016	18.8±4.8
CTD (175C-298C) Atto565-Alexa647[c]	0.027±0.011	27±11
N-C interface (52C-175C) Atto532-Alexa647[c]	0.031±0.008	24.7±13.0
N-C interface (52C-175C) Atto488-Alexa647[b]	0.035±0.020	23±3
DM-MBP (52PrK-175C-298C) Atto488-Atto565-Alexa647[c]	0.031±0.008	23±6

Table S1. The refolding rates and half-life ($t_{1/2}$) of refolding for the various MBP mutants. All the refolding measurements used for measuring the refolding rates were carried out in GuHCl concentrations below 0.1 M and the data were fitted with a mono-exponential function. The indicated error is the standard deviation from at least three independent measurements or, for the rates obtained at 25, 30 and 35° C for the NTD construct labeled with Atto532-Alexa647, the errors represent the 95% confidence intervals of the fitting error.

[a] Rate was estimated from tryptophan fluorescence measurements.

[b] Rate was estimated from the smFRET histogram by calculating the increase in the folded fraction normalized to the total refolded fraction at the end of the measurement.

[c] Rate estimated from the increase in the fraction of protein with a low acceptor lifetime relative to that of refolded protein (using Alexa647 as an acceptor). **Table S7** and **Table S8**. FRET histograms could not be used for these constructs to determine the refolding rate because of the low contrast between the intermediate population and folded state due to either the used dye pairs (in the case of CTD 175C-298C construct labeled with Atto565-Alexa647, which has a Förster distance of 70 Å), the close proximity of the attached dyes (in the case of N-C interface 52C-175C construct) or the broad FRET histogram distributions measured in 3C smFRET (in the case of DM-MBP 52PrK-175C-298C).

Table S2

	Förster distance, R_0 (Å)	Detection correction factor, γ	Spectral cross-talk, α	Direct acceptor excitation, δ	G-factor (donor / acceptor)
Atto532-Alexa647 (2C)	62	0.50/0.37*	0.03	0.07	0.85 / 0.60
Atto532-Atto647N (2C)	59	0.59	0.02	0.06	0.85 / 0.60
Atto488-Atto565 (2C / 3C)	63	0.44 / 0.40	0.07	0.05	1.15 / 1.3
Atto488-Alexa647 (2C / 3C)	53	0.25 / 0.20	0.01	0.01	1.15 / 1.25
Atto565-Alexa647 (2C / 3C)	70	0.50 / 0.43	0.14	0.13	1.3 / 1.25

Table S2. Förster distance and correction factors used for the various combinations of dye-pairs investigated in this study.

*Indicates the corrected γ used in the case of Alexa647 lifetime changes, **Fig. S4d**.

Table S3

	AV (Å)	Experimental					
		$d1$(Å)	σ_1(Å)	f_1	$d2$(Å)	σ_2(Å)	f_2
Native-NTD (52C-298C)	51.3	48.3	3.45	0.87	55.4	10	0.13
Native-CTD (175C-298C)	54.5	49.6	3.6	0.80	57.6	10	0.20
Native-N-C interface (52C-175C)	40.0	44.8	2.8	1.00	-		-
Denatured-NTD (52C-298C)	-	-	-	-	83.8	17	1.00
Denatured-CTD (175C-298C)	-	-	-	-	80.9	13	1.00
Denatured-N-C interface (52C-175C)	-	49.1	4.5	0.07	74.9	5.0	0.93

Table S3. Comparison of distances calculated from the accessible volume (AV) and 2C FRET experiments performed for all the three double-cysteine domain mutants of DM-MBP labeled with the Atto532-Alexa647 dye-pair.

Distances derived from AV simulations provide the FRET averaged distances ($\langle R_{DA} \rangle_E$) between the fluorophores and are extracted using the FPS software (*31*). Experimental distances were estimated using the photon distribution analysis (PDA) (*30*). PDA fitting was applied on the *PR* histogram obtained from the burst-wise binned raw photon counts. The Monto Carlo method was used to simulate the Gaussian distance distributions for the D-A separation with one or two populations in the *PR* histogram. The inter-dye distances, *d1* and *d2*, for the peaks of the two populations along with their respective fractions $f_1$and f_2 and widths (standard deviations) σ_1 and σ_2 are given.

Table S4

NTD (52C-298C)		τ_1 (ns)	f_1	E_1	d1 (Å)	τ_2 (ns)	f_2	E_2	d2 (Å)	χ^2_{red}
Native		0.55	0.84	0.84	46.6	2.06	0.16	0.42	65.2	1.50
Refolding in GuHCl (M)	0.1	0.58	0.79	0.83	47.2	2.14	0.21	0.40	66.1	1.61
	0.2	0.81	0.51	0.77	50.5	2.79	0.49	0.22	76.2	1.58
	0.3	0.51	0.58	0.85	45.9	2.59	0.42	0.28	72.5	1.20
	0.5	0.68	0.30	0.80	48.6	2.97	0.70	0.17	80.3	1.03
	0.9	0.47	0.38	0.86	45.3	2.92	0.62	0.18	79.2	1.07
	2	0.85	0.22	0.76	51.0	3.24	0.78	0.09	89.5	1.60
**CTD (175C-298C)**		**τ_1 (ns)**	**f_1**	**E_1**	**d1 (Å)**	**τ_2 (ns)**	**f_2**	**E_2**	**d2 (Å)**	**χ^2_{red}**
Native		0.53	0.76	0.85	46.3	1.95	0.24	0.45	63.7	1.38
Refolding in GuHCl (M)	0.1	0.47	0.66	0.86	45.2	2.36	0.34	0.34	69.1	2.03
	0.2	0.53	0.64	0.85	46.3	2.90	0.36	0.19	78.7	1.75
	0.3	0.63	0.61	0.82	47.9	3.01	0.39	0.16	81.4	1.65
	0.5	0.58	0.51	0.83	47.1	3.18	0.49	0.11	86.9	1.94
	0.9	0.56	0.45	0.84	46.8	3.15	0.55	0.12	85.8	1.95
	2	0.91	0.24	0.74	51.8	3.14	0.76	0.12	85.7	1.68
**N-C (52C-175C)**		**τ_1 (ns)**	**f_1**	**E_1**	**d1 (Å)**	**τ_2 (ns)**	**f_2**	**E_2**	**d2 (Å)**	**χ^2_{red}**
Native		0.13	0.92	0.96	36.1	2.29	0.08	0.36	68.1	1.21
Refolding in GuHCl (M)	0.1	0.49	0.74	0.86	45.7	2.38	0.26	0.33	69.3	1.63
	0.2	0.62	0.69	0.82	47.7	2.62	0.31	0.27	73.0	1.85
	0.3	0.54	0.59	0.84	46.5	2.61	0.41	0.27	72.9	1.74
	0.5	0.80	0.56	0.77	50.3	2.58	0.44	0.28	72.4	1.62
	0.9	0.35	0.59	0.90	42.8	2.49	0.41	0.30	71.0	1.44
	2	0.65	0.23	0.81	48.3	2.95	0.77	0.17	79.9	1.71

Table S4. Results from the donor lifetime analysis of all three 2C constructs of DM-MBP labeled with Atto532-Alexa647.

The donor (Atto532) fluorescence lifetimes (τ_1 and τ_2), their fractions (f_1 and f_2), the corresponding calculated FRET efficiencies (E1 and E2) and the distance estimations (d1 and

d2) from a biexponential fit of the fluorescence decay for different double-cysteine mutants of DM-MBP labeled with Atto532-Alexa647 are given. For lifetime determination, all photons coming from double-labeled bursts that meet the selection criteria (Material and Methods) are summed together and the TCSPC histogram fit using a biexponential function. For comparison, the lifetime fractions from the native state and under denaturing conditions are given. Refolding was measured at the given GuHCl concentrations. All fits are given in **Fig. S6**.

Table S5

NTD (52C-298C)		τ_{R1} (μs)	f_1	τ_{R2} (μs)	f_2
Refolding in GuHCl (M)	0.3	9.7±3.4	0.57±0.21	175±56	0.43±0.09
	0.5		0.67±0.22		0.33±0.08
	0.9		0.68±0.28		0.32±0.11
CTD (175C-298C)		τ_{R1} (μs)	f_1	τ_{R2} (μs)	f_2
Refolding in GuHCl (M)	0.2	7.6±0.9	0.69±0.08	274±36	0.31±0.05
	0.3		0.60±0.24		0.40±0.06
	0.5		0.64±0.14		0.36±0.04
	0.9		0.54±0.09		0.46±0.03
N-C (52C-175C)		τ_{R1} (μs)	f_1	τ_{R2} (μs)	f_2
Refolding in GuHCl (M)	0.2	3.6±0.8	0.35±0.23	347±35	0.65±0.09
	0.3		0.67±0.23		0.33±0.14
	0.5		0.44±0.20		0.56±0.03
	0.9		0.70±0.11		0.30±0.07

Table S5. Results from the filtered FCS analysis for all the three 2C constructs of DM-MBP labeled with Atto532-Alexa647.

From the filtered FCS analysis, two relaxation times, given as τ_{R1} and τ_{R2}, were visible and their respective fractions are given as f_1 and f_2 respectively (**Fig. S11**). Errors indicate the 95% confidence interval from the fit assessed using the χ^2_{red}. The kinetics rates for the GuHCl concentration measurements of 0.2, 0.3, 0.5 and 0.9 for each construct were fit globally (with the exception of the NTD) whereas the amplitudes were allowed to vary. For the 0.2 M GuHCl measurements of the NTD, fluctuations of the signal were not visible as half of the molecules are in a near native-like state (**Fig. 1**). Native and 2 M GuHCl could not be analyzed due to low contrast in the smFRET histogram for selecting the sub-populations.

Table S6

DM-MBP NTD (52C-298C)		R_C (Å)	$k_{C\to U}$ (ms^{-1})	R_U (Å)	$k_{U\to C}$ (ms^{-1})	τ_R (μs)
Refolding in GuHCl (M)	0.1	47.2	0.12±0.03	66.1	0.72±0.19	1190±287
	0.2	45.9*	1.03±0.10	76.2	3.23±0.27	234±15
	0.3	45.9	1.38±0.12	72.5	2.13±0.22	284±19
	0.5	48.6	2.08±0.21	80.3	0.89±0.10	336±26
	0.9	45.3	1.91±0.17	79.2	0.63±0.07	393±28
DM-MBP CTD (175C-298C)		R_C (Å)	$k_{C\to U}$ (ms^{-1})	R_U (Å)	$k_{U\to C}$ (ms^{-1})	τ_R (μs)
Refolding in GuHCl (M)	0.1	45.2	1.19±0.09	69.1	2.19±0.17	295±16
	0.2	46.3	1.02±0.11	78.7	1.98±0.37	333±43
	0.3	47.9	1.17±0.11	81.4	2.76±0.22	254±15
	0.5	47.1	2.31±0.22	81.4*	3.00±0.28	188±12
	0.9	46.8	3.95±0.29	81.4*	1.60±0.12	180±10
DM-MBP N-C (52C-175C)		R_C (Å)	$k_{C\to U}$ (ms^{-1})	R_U (Å)	$k_{U\to C}$ (ms^{-1})	τ_R (μs)
Refolding in GuHCl (M)	0.1	44.8*	0.13±0.04	69.3	0.62±0.16	1333±307
	0.2	44.8*	0.39±0.05	73.0	3.34±0.59	268±43
	0.3	46.5	0.77±0.07	72.9	3.85±0.33	216±16
	0.5	44.8*	0.55±0.08	72.4	3.75±0.49	232±27
	0.9	46.5*	1.70±0.23	71.0	3.51±0.55	191±22
DM-MBP NTD (52C-298C)		R_C (Å)	$k_{C\to U}$ (ms^{-1})	R_U (Å)	$k_{U\to C}$ (ms^{-1})	τ_R (μs)
Refolding in GuHCl (M) + TMAO	0.2	45.9	0.43±0.05	76.2	2.50±0.27	340±32
WT-CTD (175C-298C)		R_C (Å)	$k_{C\to U}$ (ms^{-1})	R_U (Å)	$k_{U\to C}$ (ms^{-1})	τ_R (μs)
Refolding in GuHCl (M)	1	47.9	2.34±0.21	79.5	2.57±0.3	203±19

Table S6. Results from the dynamic PDA for all the three 2C DM-MBP constructs labeled with Atto532-Alexa647.

Data for refolding measurements performed in 0.1, 0.2, 0.3, 0.5, and 0.9 M GuHCl were analyzed for the 2C FRET measurements on the NTD, CTD and N-C interface constructs of DM-MBP. In addition, the DM-MBP NTD construct was analyzed when refolding in the presence of 0.2 M GuHCl supplemented with 500 mM TMAO. The CTD construct of WT-MBP was measured in 1 M GuHCl and also analyzed with dynamic PDA. By fixing the two inter-dye distances for the compact and unfolded states (R_C and R_U respectively) estimated from the lifetime analysis (**Table S4**), the microscopic rates for interconversion ($k_{U\text{->}C}$ and $k_{C\text{->}U}$) between the states could be extracted. Widths were fixed to a constant fraction of the dye separation *R* (0.07 *R*) (*32*). The relaxation time, τ_R, was calculated as 1/($k_{U\text{->}C}$ + $k_{C\text{->}U}$).

* In the marked cases, distances used in the dynamic PDA analysis were extracted from the FRET efficiencies measured in the intermediate GuHCl titrations or from the native state because the fluorescence lifetime values at very high and very low FRET efficiencies can be difficult to determine correctly (e.g. due to donor and acceptor quenching and/or donor only/acceptor blinking events). The given errors represent the 95% confidence intervals obtained from the Jacobian matrix upon fitting as assessed by the χ^2_{red}. **Fig. S10**.

Table S7

		τ_1 (ns)	f_1	τ_2 (ns)	f_2
N-C interface (52C-175C) Atto532-Alexa647	Native	1.34	1	-	-
	Refolding, initial 5 min	-	-	1.67	1
	Refolded	1.29	0.75	1.86	0.25
	Denatured	1.51	1	-	-
CTD (175C-298C) Atto565-Alexa647	Native	1.43	1	-	-
	Refolding, initial 5 min	1.44	0.03	1.80	0.97
	Refolded	1.44	0.48	1.80	0.52
	Denatured	1.60	1	-	-

Table S7. Fluorescence lifetime analysis of Alexa647 for two-color DM-MBP constructs. The fluorescence lifetime of DM-MBP constructs containing Alexa647 as an acceptor were fit with a single- or double-exponential function. The resulting lifetimes and their corresponding fractions (f) for the various of DM-MBP constructs are given. The double-cysteine mutants were stochastically labeled with the respective dyes. (**Fig. S4a**).

Table S8

		τ_1 (ns)	f_1	τ_2 (ns)	f_2
DM-MBP (52PrK-175C-298C)	Native	1.33	0.77	1.74	0.23
	Refolding, initial 15 min	1.25	0.23	1.75	0.77
	Refolded	1.25	0.62	1.75	0.38
	Denatured	1.48	1	-	-

Table S8. Fluorescence lifetimes of Alexa647 for triple-labeled molecules of DM-MBP (52PrK-175C-298C).

The fluorescence lifetime of 3C DM-MBP containing Alexa647 as an acceptor was fit with a single- or double-exponential function. The determined fluorescence lifetime and respective fractions (f) are given. This construct was specifically labeled with Atto488 (at position 52), Atto565 (at position 298) and Alexa647 (at position 175) (**Fig. 1a**).

Table S9

DM-MBP		Donor (Atto532)		Acceptor (Alexa647)	
NTD (52C-298C)		$\langle r \rangle$	r_∞	$\langle r \rangle$	r_∞
Native		0.249	0.059	0.196	0.121
	0.1	0.207	0.038	0.213	0.213
	0.2	0.168	0.014	0.227	0.165
Refolding in	0.3	0.186	0.047	0.226	0.178
GuHCl (M)	0.5	0.113	0.001	0.225	0.145
	0.9	0.147	0.014	0.218	0.165
	2	0.086	0.001	0.182	0.062
	0.1	0.269	0.015	0.191	0.191
	0.2	0.231	0.070	0.248	0.240
Unfolding in	0.3	0.221	0.020	0.199	0.195
GuHCl (M)	0.5	0.211	0.062	0.194	0.167
	0.9	0.147	0.022	0.285	0.225
	2	0.072	0.001	0.191	0.170
DM-MBP		Donor (Atto532)		Acceptor (Alexa647)	
CTD (175C-298C)		$\langle r \rangle$	r_∞	$\langle r \rangle$	r_∞
Native		0.246	0.068	0.246	0.109
	0.1	0.213	0.033	0.239	0.148
	0.2	0.178	0.014	0.242	0.178
Refolding in	0.3	0.163	0.011	0.241	0.183
GuHCl (M)	0.5	0.140	0.011	0.233	0.173
	0.9	0.126	0.004	0.236	0.169
	2	0.122	0.003	0.238	0.163
	0.1	0.237	0.068	0.224	0.167
	0.2	0.236	0.064	0.220	0.169
Unfolding in	0.3	0.234	0.052	0.219	0.166
GuHCl (M)	0.5	0.234	0.051	0.223	0.170
	0.9	0.202	0.029	0.231	0.172
	2	0.122	0.010	0.204	0.121

Table S9 (Con't)

DM-MBP		Donor (Atto532)		Acceptor (Alexa647)	
N-C (52C-175C)		$\langle r \rangle$	r_∞	$\langle r \rangle$	r_∞
Native		0.185	0.034	0.209	0.200
	0.1	0.193	0.015	0.239	0.188
	0.2	0.198	0.045	0.241	0.217
Refolding in	0.3	0.197	0.047	0.258	0.201
GuHCl (M)	0.5	0.182	0.033	0.230	0.211
	0.9	0.143	0.021	0.229	0.200
	2	0.127	0.012	0.235	0.115
	0.1	0.203	0.051	0.193	0.190
	0.2	0.200	0.040	0.198	0.189
Unfolding in	0.3	0.195	0.040	0.208	0.199
GuHCl (M)	0.5	0.200	0.033	0.202	0.201
	0.9	0.160	0.016	0.212	0.190
	2	0.160	0.016	0.203	0.148

Table S9. Steady-state and time-resolved anisotropies of DM-MBP constructs of NTD, CTD and N-C interface measured with Atto532 and Alexa647.

The steady-state $\langle r \rangle$ and residual anisotropy r_∞ were calculated for double-labeled molecules from the 2C smFRET measurements by summing together the photons coming from all dual-color bursts that meet the selection criteria (**Material and Methods**). For the steady-state anisotropy, only the polarization channel information was used. For the time-resolved anisotropy analysis, the TCSPC information was also included. The time-resolved anisotropy data were then approximated using a single exponential function to extract the residual anisotropy (see **Supplementary Note 3**). The G factor to compensate for different sensitivities of the parallel and perpendicular detection channels are given in (**Table S2**).

Source Data files: Unprocessed SDS gel images (Figure S12)

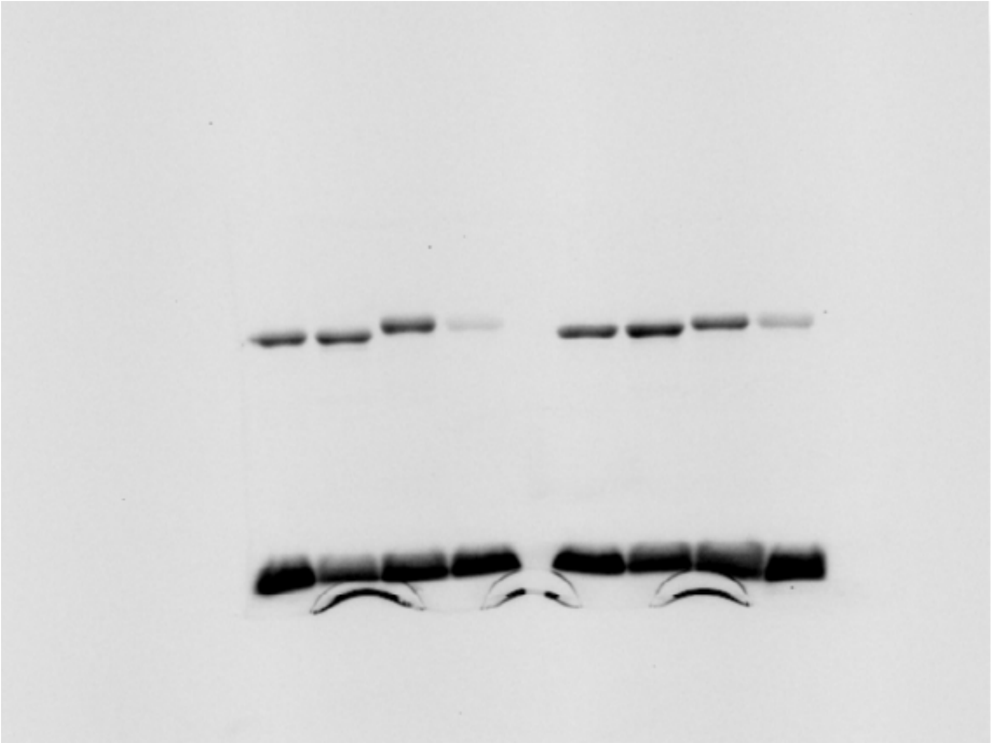

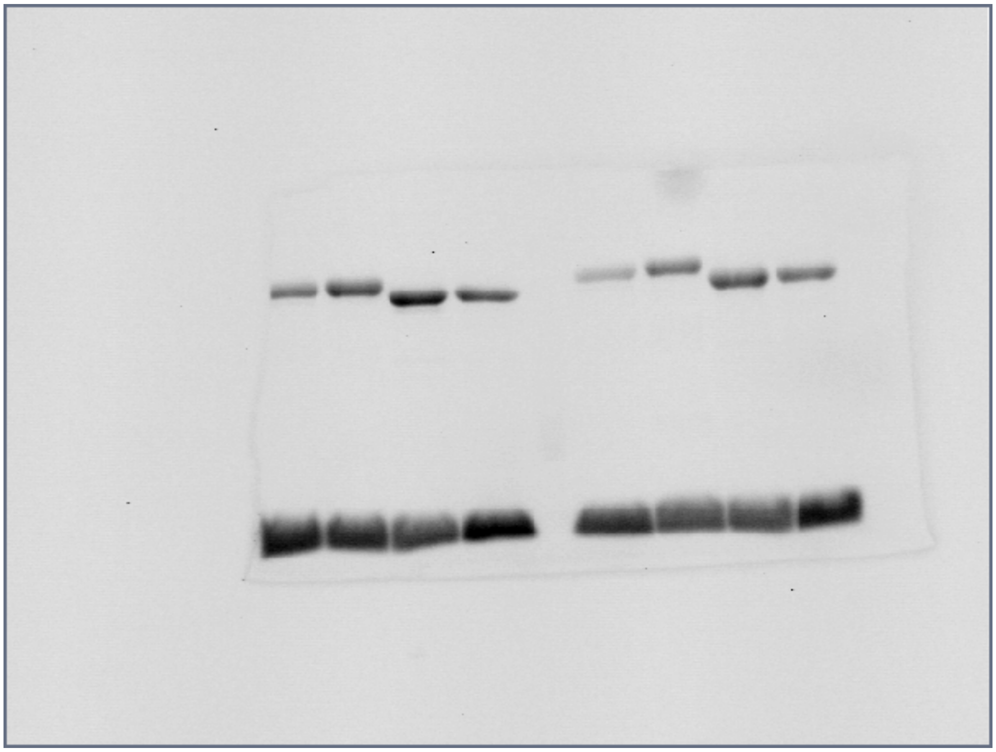

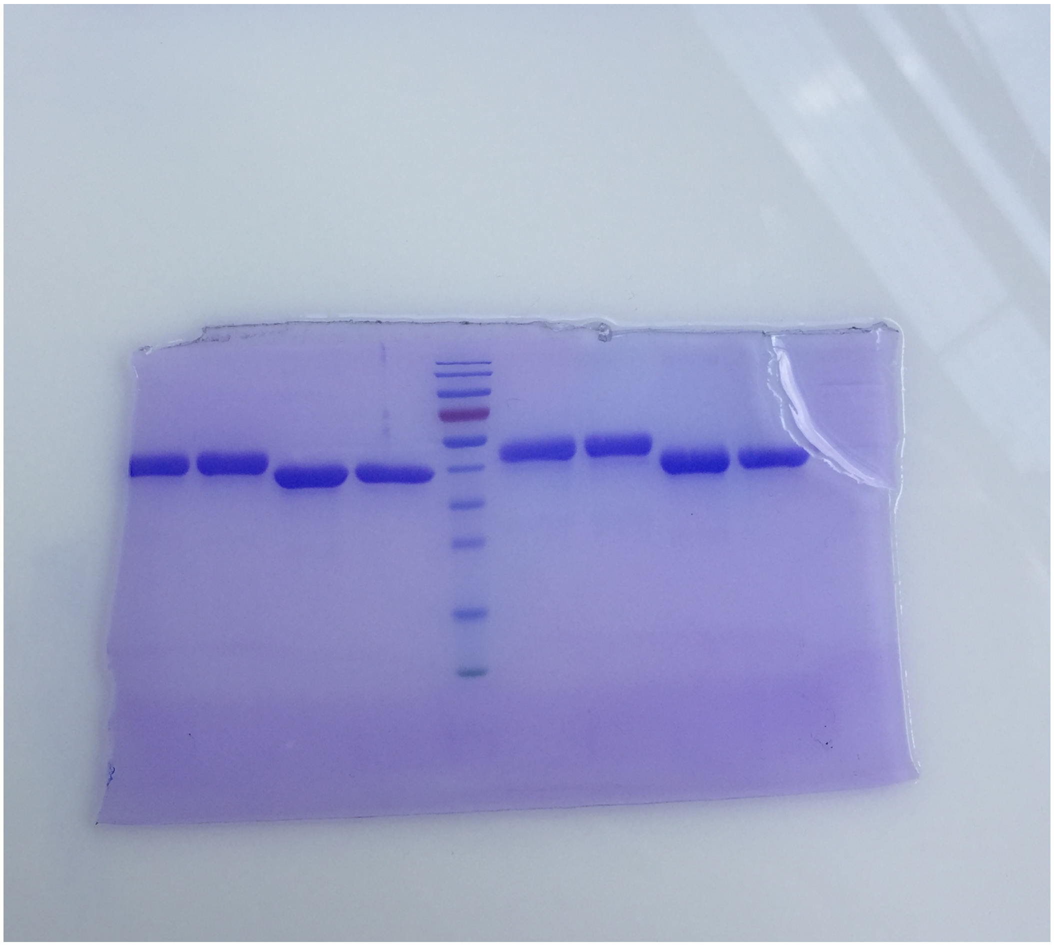

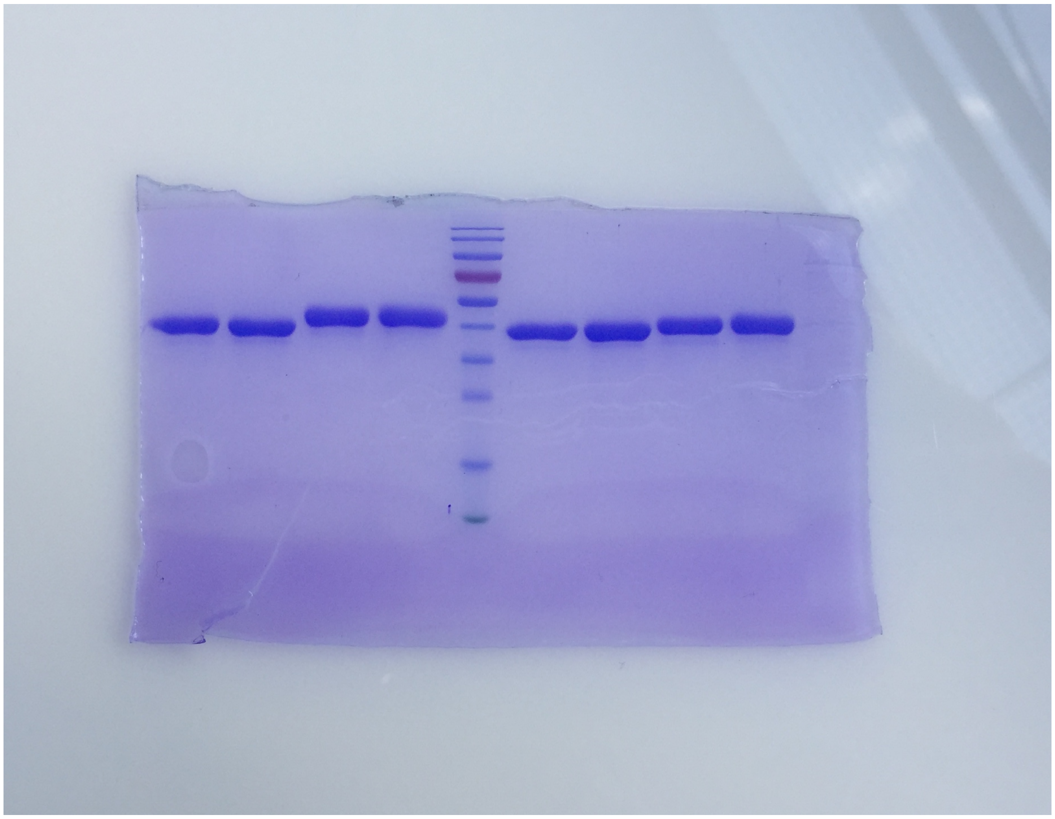

Supplement: Supplementary file 1 — Supplementary Information [file 41467_2024_44901_MOESM1_ESM.pdf]
