# Supplementary material for: Folding pathway of a discontinuous two-domain protein
Source: Nat Commun. 2024 Jan 23;15:690. doi: 10.1038/s41467-024-44901-3 (PMC10805907; doi:10.1038/s41467-024-44901-3)
Supplement: Supplementary file 2 — Reporting Summary [file 41467_2024_44901_MOESM2_ESM.pdf]

nature portfolio

Corresponding author(s): Don C. Lamb

Last updated by author(s): 14.12.2023

Reporting Summary

Nature Portfolio wishes to improve the reproducibility of the work that we publish. This form provides structure for consistency and transparency in reporting. For further information on Nature Portfolio policies, see our Editorial Policies and the Editorial Policy Checklist.

Statistics

For all statistical analyses, confirm that the following items are present in the figure legend, table legend, main text, or Methods section.

n/a	Confirmed	
☐	☒	The exact sample size (*n*) for each experimental group/condition, given as a discrete number and unit of measurement
☐	☒	A statement on whether measurements were taken from distinct samples or whether the same sample was measured repeatedly
☒	☐	The statistical test(s) used AND whether they are one- or two-sided<br>*Only common tests should be described solely by name; describe more complex techniques in the Methods section.*
☒	☐	A description of all covariates tested
☒	☐	A description of any assumptions or corrections, such as tests of normality and adjustment for multiple comparisons
☐	☒	A full description of the statistical parameters including central tendency (e.g. means) or other basic estimates (e.g. regression coefficient) AND variation (e.g. standard deviation) or associated estimates of uncertainty (e.g. confidence intervals)
☒	☐	For null hypothesis testing, the test statistic (e.g. *F*, *t*, *r*) with confidence intervals, effect sizes, degrees of freedom and *P* value noted<br>*Give P values as exact values whenever suitable.*
☒	☐	For Bayesian analysis, information on the choice of priors and Markov chain Monte Carlo settings
☒	☐	For hierarchical and complex designs, identification of the appropriate level for tests and full reporting of outcomes
☒	☐	Estimates of effect sizes (e.g. Cohen's *d*, Pearson's *r*), indicating how they were calculated

Our web collection on statistics for biologists contains articles on many of the points above.

Software and code

Policy information about availability of computer code

Data collection	All the single molecule fluorescence data was collected with SPCM software (Becker and Hickl) or HydrHarp400(PicoQaunt). All the tryptophan fluorescence measurements performed on FLS1000 Photoluminescence Spectrometer (Edinburgh Instruments) were collected with Fluoracle Software.
Data analysis	All the single molecule fluorescence data was analyzed with PAM (PIE analysis with Matlab) software package (Ref.70). sff14SB forcefield (The AMBER16 MD package) was used for MD simulations. Statistical data was analyzed with Origin 10.0 and accessible volume calculations with FRET positioning and screening (FPS) software (Ref.29).

For manuscripts utilizing custom algorithms or software that are central to the research but not yet described in published literature, software must be made available to editors and reviewers. We strongly encourage code deposition in a community repository (e.g. GitHub). See the Nature Portfolio guidelines for submitting code & software for further information.

Data

Policy information about availability of data

All manuscripts must include a data availability statement. This statement should provide the following information, where applicable:

- Accession codes, unique identifiers, or web links for publicly available datasets
- A description of any restrictions on data availability
- For clinical datasets or third party data, please ensure that the statement adheres to our policy

Raw data for the main figures Figure 1B-C, Figure 2A, C-D, Figure 4B-C, Figure 5B, C-D and for the supplementary Figures Figure S1A-B, Figure S3C, E, G, Figure S5,

Figure S7A-B, Figure S8A, D, Figure S9, and Figure S15A-C. Figure S17 are deposited on zenodo https//doi.org/10.5281/zenodo.8007031 (part I) and https://doi.org/10.5281/zenodo.10407188 (Part II). PDB ID used :1OMP.

Research involving human participants, their data, or biological material

Policy information about studies with human participants or human data. See also policy information about sex, gender (identity/presentation), and sexual orientation and race, ethnicity and racism.

Reporting on sex and gender	n/a
Reporting on race, ethnicity, or other socially relevant groupings	n/a
Population characteristics	n/a
Recruitment	n/a
Ethics oversight	n/a

Note that full information on the approval of the study protocol must also be provided in the manuscript.

Field-specific reporting

Please select the one below that is the best fit for your research. If you are not sure, read the appropriate sections before making your selection.

[x] Life sciences [ ] Behavioural & social sciences [ ] Ecological, evolutionary & environmental sciences

For a reference copy of the document with all sections, see nature.com/documents/nr-reporting-summary-flat.pdf

Life sciences study design

All studies must disclose on these points even when the disclosure is negative.

Sample size	One FRET mutant of WT-MBP protein and 4 FRET mutants (including 3 mutants for 2 color and one mutant for 3 color) of Double mutant (DM)- MBP protein. Typical measurements contained at least x,000 burst. Our experience indicates that this is sufficient statistics for the types of single molecule FRET analysis performed here.
Data exclusions	No data was excluded from the analysis.
Replication	Measurements were replicated as mentioned in the figure legends as applicable.
Randomization	As the single molecules are randomly sampled in the experiment, there is no additional advantage to randomizing the data.
Blinding	The results expected from the measurements was unknown. Hence, there was no advantage to the user in knowing what sample was being measured.

Reporting for specific materials, systems and methods

We require information from authors about some types of materials, experimental systems and methods used in many studies. Here, indicate whether each material, system or method listed is relevant to your study. If you are not sure if a list item applies to your research, read the appropriate section before selecting a response.

Materials & experimental systems

n/a	Involved in the study	
[x]	[ ]	Antibodies
[x]	[ ]	Eukaryotic cell lines
[x]	[ ]	Palaeontology and archaeology
[x]	[ ]	Animals and other organisms
[x]	[ ]	Clinical data
[x]	[ ]	Dual use research of concern
[x]	[ ]	Plants

Methods

n/a	Involved in the study	
[x]	[ ]	ChIP-seq
[x]	[ ]	Flow cytometry
[x]	[ ]	MRI-based neuroimaging

Plants

Seed stocks	*Report on the source of all seed stocks or other plant material used. If applicable, state the seed stock centre and catalogue number. If plant specimens were collected from the field, describe the collection location, date and sampling procedures.*
Novel plant genotypes	*Describe the methods by which all novel plant genotypes were produced. This includes those generated by transgenic approaches, gene editing, chemical/radiation-based mutagenesis and hybridization. For transgenic lines, describe the transformation method, the number of independent lines analyzed and the generation upon which experiments were performed. For gene-edited lines, describe the editor used, the endogenous sequence targeted for editing, the targeting guide RNA sequence (if applicable) and how the editor was applied.*
Authentication	*Describe any authentication procedures for each seed stock used or novel genotype generated. Describe any experiments used to assess the effect of a mutation and, where applicable, how potential secondary effects (e.g. second site T-DNA insertions, mosiacism, off-target gene editing) were examined.*
